# Chromosome Evolution in Amniote Vertebrates: A Comprehensive Atlas of Karyotypic Diversity From Ancestral States to Rapid Turnover

**DOI:** 10.1111/mec.70608

**Published:** 2026-11-07

**Authors:** Martin Knytl, Tiago Marafiga Degrandi, Dmitry Prokopov, Vladimir Trifonov, Lívia do Vale Martins, Francisco de Menezes Cavalcante Sassi, Marcelo Ricardo Vicari, Marcelo de Bello Cioffi, Marcello Mezzasalma

**Affiliations:** ^1^ Department of Cell Biology Charles University Prague Czech Republic; ^2^ Department of Biology McMaster University Hamilton Ontario Canada; ^3^ Laboratory of Genetics and Evolution, Department of Structural, Molecular Biology and Genetics State University of Ponta Grossa Ponta Grossa Brazil; ^4^ Centre for Haemato‐Oncology, Barts Cancer Institute Queen Mary University of London London UK; ^5^ QMUL Centre for Epigenetics Queen Mary University of London London UK; ^6^ Institute of Animal Physiology and Genetics CAS Liběchov Czech Republic; ^7^ Federal University of Piauí Floriano Brazil; ^8^ Post‐Graduation Program in Agronomy Federal University of Piauí Teresina Brazil; ^9^ MOE Key Laboratory of Freshwater Fish Reproduction and Development, School of Life Sciences Southwest University Chongqing China; ^10^ Aquaculture Center of UNESP São Paulo State University Jaboticabal Brazil; ^11^ Department of Structural Biology, Molecular and Genetics Universidade Estadual de Ponta Grossa Ponta Grossa Brazil; ^12^ Departamento de Genética e Evolução Universidade Federal de São Carlos São Carlos Brazil; ^13^ Department of Biology, Ecology and Earth Science University of Calabria Rende Italy

**Keywords:** ancestral karyotype, birds, genome evolution, mammals, non‐avian reptiles

## Abstract

The evolution of the amniote genome is characterized by a striking dichotomy between extreme karyotypic stasis and rapid structural innovation. To identify the mechanisms governing this variation, we provide a comprehensive synthesis of chromosome number and karyotype data across non‐avian reptiles, birds and mammals, integrating a century of classical cytogenetics with modern chromosome‐scale genomics. Amniote chromosome numbers span a range from 2*n* = 6 to 142 and have been shaped primarily by chromosomal fusions and fissions rather than by polyploidization common in non‐amniotes. Recent ancestral state reconstructions suggest a highly fragmented ancestral amniote karyotype of 2*n* = 88 with a bipartite organization of macro‐ and microchromosomes. They also support environmental sex determination as the plesiomorphic state. We explore the evolutionary mechanisms facilitating chromosomal turnover, including lineage‐specific transposable element activity, hybridization‐driven genomic instability and genetic hitchhiking, alongside the canalization model and the position‐effect hypothesis as primary responses to environmental transitions. Social structure, such as polygynous harem systems, can further contribute to rearrangement fixation via genetic drift in some, but not all, lineages. Chromosomal divergence across these lineages serves as a useful cytotaxonomic marker and, in some cases, a barrier to gene flow, as in the postzygotic incompatibility of equid and cervid hybrids. In contrast, introgression persists across karyotypically divergent rock‐wallabies and crocodilians, indicating that the reproductive consequences of karyotypic divergence are lineage‐specific rather than a fixed function of karyotypic distance. This synthesis presents an updated atlas of amniote chromosomal evolution and provides a conceptual framework for understanding how molecular mechanisms, ecological shifts and behavioural factors interact to shape genomic structure.

## 1 Introduction

Since diverging from amphibians approximately 350 million years ago (Mya) (Kumar et al. 2022), the lineages leading to modern reptiles, birds and mammals have followed distinct trajectories of genomic organization. While many birds and non‐avian reptiles have maintained a highly conserved ancestral karyotype containing large macrochromosomes and numerous tiny microchromosomes, most mammalian lineages have undergone extensive reorganization, leading to the loss of microchromosomes and the emergence of highly variable chromosome numbers (Damas et al. 2022; Zhang et al. 2014). These architectural shifts, alongside the diverse evolution of sex‐determining systems, provide a framework for reconstructing ancestral characteristics and for testing how genomic and ecological changes have driven speciation and biological complexity across the amniote phylogeny (Deakin and Ezaz 2014). To characterize this variation, from karyotypic stasis to rapid structural innovation, we compiled comprehensive datasets on chromosome numbers in amniote vertebrates from peer‐reviewed articles, reviews, books and online karyotype and genome databases using keywords: ‘chromosome number’, ‘karyotype’ and ‘cytogenetics’ paired with the appropriate vertebrate taxa.

## 2 Non‐Avian Reptiles

This group comprises crocodilians, chelonians (turtles and tortoises) and lepidosaurs—squamates (lizards and snakes) and the tuatara (*Sphenodon*). These three lineages have evolved independently for over 250 million years (My). Given this deep phylogenetic separation and the resulting differences in their genomic architecture, we constructed three separate phylogenetic trees to ensure accurate, clade‐specific reconstructions of chromosomal evolution.

### 2.1 Crocodilians

The order Crocodylia includes 28 extant archosaurian species subdivided into three families: Gavialidae (two genera and two species), Alligatoridae (four genera and eight species) and Crocodylidae (three genera and 18 species) (Figure 1; Uetz et al. 2023). Phylogenetic reconstructions of the modern crocodilians using fossil and molecular data alternatively place Gavialidae and Alligatoridae as the most basal clades, while both approaches recognize the family Crocodylidae as the most recently diverged group (see Brochu 2003; Colston et al. 2020; Oaks 2011). However, it should be noted that the modern crocodilians represent a small semiaquatic fraction of a much larger and more diversified clade of extinct archosaurs and that the evolution of the extant clades is believed to have remained ecologically and morphologically highly stable for about 100 My (e.g., Mannion et al. 2019; Stockdale and Benton 2021).

**FIGURE 1:**
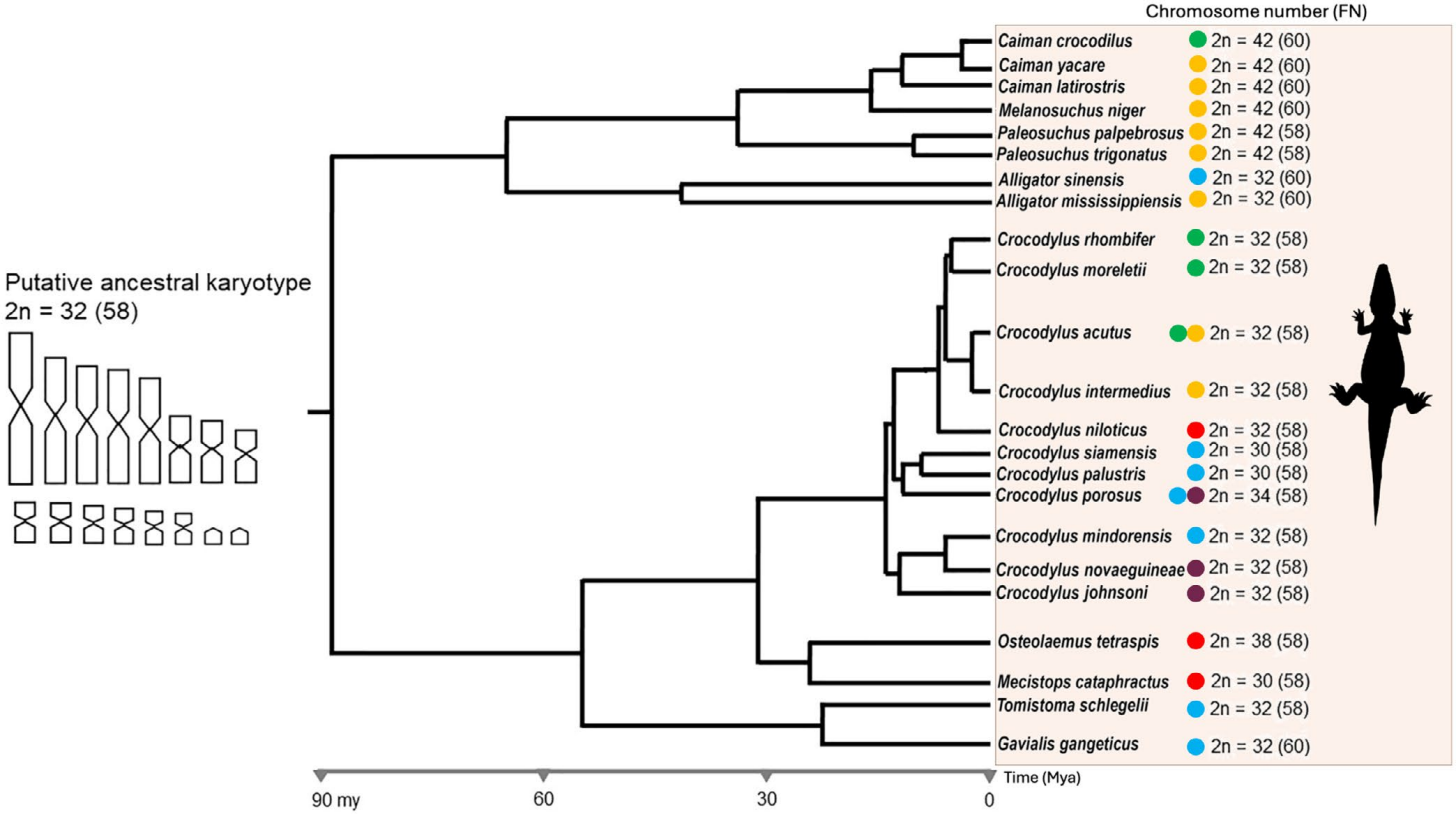
Phylogeny of crocodilians illustrating the evolution of diploid chromosome number. The known range of diploid (2*n*) chromosome numbers and fundamental number (FN) in parentheses are indicated for each species. Putative ancestral karyotype shows 32 chromosomes and FN of 58 containing 14 pairs of metacentric and two pairs of acrocentric chromosomes. Sex chromosomes are not depicted since temperature‐dependent sex determination is considered the ancestral condition. The *Paleosuchus palpebrosus* silhouette represents Crocodylia. Phylogenetic relationships follow Uetz et al. (2023). Coloured circles represent continent of occurrence, categorized as North and Central America (green), South America (orange), Africa (red), Asia (sky blue) and Oceania (purple).

From a cytogenetic point of view, the majority of the extant crocodilians (23 out of the 28 currently described species) have been studied using standard karyotyping, banding techniques and/or molecular cytogenetics (Cohen and Gans 1970; Oliveira et al. 2021; Srikulnath, Thapana, et al. 2015). Overall, the whole order is characterized by a karyotype composed of 2*n* = 30–42 chromosomes (Figure 1; Table S1), with a variable number of biarmed and uniarmed (acrocentric) elements, gradually decreasing in length without any clear distinction between macro‐ and microchromosomes (e.g., Cohen and Gans 1970; Oliveira et al. 2021). There are no known sex chromosomes in Crocodylia and all extant species exhibit temperature‐dependent sex determination (TSD) (González et al. 2019).

Overall, all crocodilians show a fundamental arm number (FN) of 56–60 and no variability in the chromosome number within genera, with the sole exception of *Crocodylus*. In Gavialidae, *Gavialis gangeticus* and *Tomistoma schlegelii* share a very similar karyotype of 2*n* = 32, but with a different number of metacentric/uniarmed pairs (Cohen and Gans 1970; Srikulnath, Thapana, et al. 2015). In Alligatoridae, *Alligator mississippiensis* and *A. sinensis* share the same karyotype of 2*n* = 32 elements, while the genera *Caiman*, *Melanosuchus* and *Paleosuchus* (collectively representing the sister clade to *Alligator*) all show a karyotype composed of 2*n* = 42, with a higher number of acrocentric elements and a lower number of biarmed chromosomes (Oliveira et al. 2021; Srikulnath, Thapana, et al. 2015). In Crocodylidae, the 11 species of the genus *Crocodylus* which have been karyologically investigated show a variable chromosome complement of 2*n* = 30–34, with the most common chromosome number of 2*n* = 32 occurring in eight species (Figure 1). Interestingly, the karyotype of these species is very similar to the 2*n* = 32 found in Gavialidae and *Alligator*, with five large metacentric pairs, several medium‐sized biarmed elements and one to three acrocentric pairs (Cohen and Gans 1970; Oliveira et al. 2021; Srikulnath, Thapana, et al. 2015). Additionally, *Mecistops cataphractus* and *Osteolaemus tetraspis* , which belong to a monophyletic group representing the sister clade to *Crocodylus*, show a different karyotype composed of 2*n* = 30 and 2*n* = 38, respectively (Cohen and Gans 1970; Oliveira et al. 2021; Srikulnath, Thapana, et al. 2015).

Interestingly, the chromosome number in Crocodylia is positively correlated with the number of acrocentric chromosomes, suggesting that their karyotype evolution has been mostly driven by fusion and fission events (Srikulnath, Thapana, et al. 2015), by which the number of chromosomes decreases and increases, respectively. Starting from this observation, two alternative hypotheses on the chromosome evolution in the order Crocodylia can be proposed by taking into account the available cytogenetic data (Cohen and Gans 1970; Oliveira et al. 2021; Srikulnath, Thapana, et al. 2015). The first hypothesis indicates a karyotype with a relatively high chromosome number (e.g., the 2*n* = 42 found in *Caiman*, *Melanosuchus* and *Paleosuchus*) as the ancestral condition of the group and chromosome fusions as the main rearrangements involved in the progressive reduction of the chromosome number observed in different evolutionary lineages. However, the opposite hypothesis involving a progressive increase in chromosome number starting from an ancestral karyotype of 2*n* = 32 is probably the most likely and parsimonious chromosomal evolutionary scenario in Crocodylia. In fact, a karyotype composed of 2*n* = 32, with five large metacentric pairs is the only karyotype that occurs in different species of all the three extant families. Furthermore, the karyotypes with a relatively low chromosome count which have been analysed with molecular cytogenetics did not show any interstitial telomeric repeats (ITRs), which are usually interpretable as good indicators of past or recent chromosome fusions and/or inversions (e.g., Lin and Yan 2008; Vicari et al. 2022).

In Gavialidae, *G. gangeticus* and *T. schlegelii* share a karyotype of 2*n* = 32, but with a different FN (58 and 60, respectively) likely due to a chromosome inversion or centromere repositioning which drove the transition between biarmed and uniarmed chromosome pairs (Srikulnath, Thapana, et al. 2015).

In Alligatoridae, *A. sinensis* and *A. mississippiensis* show the same karyotype as *G. gangeticus* of 2*n* = 32 with five large metacentric pairs, nine medium‐sized biarmed pairs and two acrocentric pairs, which has therefore been hypothesized as the ancestral chromosome complement in crocodilians. Consequently, the higher chromosome count found in *Caiman*, *Paleosuchus* and *Melanosuchus* (2*n* = 42) likely originated from five centric fissions (Srikulnath, Thapana, et al. 2015).

Starting from the same hypothesized ancestral karyotype of 2*n* = 32, both reduction and increase in the chromosome number can be observed in Crocodylidae. In particular, the karyotype of *M. cataphractus* with a lower chromosome number and a higher number of biarmed chromosomes (2*n* = 30, FN = 58), likely originated from centric fusions between ancestral acrocentric elements. On the other hand, the higher diploid chromosome number with a higher number of acrocentric chromosomes of *O. tetraspis* (2*n* = 38) likely derived from the ancestral 2*n* = 32 by means of multiple centric fissions (Srikulnath, Thapana, et al. 2015). In *Crocodylus*, multiple chromosome evolutionary pathways can be observed in different lineages, including the conservation of karyotypes similar to that of hypothesized ancestral 2*n* = 32 (in eight species), the reduction of the chromosome number via centric fusions which likely gave rise to the 2*n* = 30 of *C. rhombifer* , *C. siamensis* and *C. palustris* , and lastly, the increase in the total chromosome number to the 2*n* = 34 of *C. porosus* via a single centric fission. It should be noted that the karyotypes of 2*n* = 32 of most *Crocodylus* species show a different FN when compared to the hypothesized ancestral condition (58 and 60, respectively), which is likely due to a single chromosome inversion (Srikulnath, Thapana, et al. 2015).

Interestingly, despite differences in chromosome number, the congeneric *C. siamensis* (2*n* = 30) and *C. porosus* (2*n* = 34) are known to produce viable F1 hybrids with 2*n* = 32, and F2 offspring have also been reported, indicating that chromosomal divergence does not necessarily impose strong postzygotic barriers in this lineage (Srikulnath, Thapana, et al. 2015). Thus, although karyotypic differences have been proposed to contribute to lineage diversification in crocodilians, available evidence suggests that their effects on reproductive isolation may be limited, particularly within the relatively recent radiation of *Crocodylus* (Srikulnath, Thapana, et al. 2015). At a broader phylogenetic scale, the karyotypic differences of the major crocodilian lineages correspond to their geographic and phylogenetic structure. In particular, the integration of phylogenetic relationships, geographic distribution and variation in diploid chromosome number suggests that increases in chromosome number occurred independently in several extant crocodilian lineages, namely in Central and South American Alligatoridae (*Caiman*, *Melanosuchus* and *Paleosuchus*) and in Asian‐Oceanian ( *C. porosus* ) and African ( *O. tetraspis* ) Crocodylidae (Figure 1), a pattern consistent with chromosome evolution accompanying their independent diversification.

### 2.2 Squamates and *Sphenodon*

The New Zealand tuatara ( *Sphenodon punctatus* ) is the only extant species of the order Rhynchocephalia, whose representatives were globally distributed until the end of the Mesozoic (Jones 2008; Uetz et al. 2023). The taxonomic status of these living populations has also been recently refined. Although two species, *S. punctatus* and the rarer *S. guntheri* , were historically recognized, comprehensive molecular analyses have since revised this view. Current taxonomy now recognizes only a single tuatara species, which comprises three divergent and geographically distinct genetic clades (Hay et al. 2010).

Based on morphological and molecular evidence, the order Rhynchocephalia is recognized as the sister group to Squamata and was estimated to diverge from their lepidosaurian ancestor around 240–270 Mya (Figure 2; Simões et al. 2020).

**FIGURE 2:**
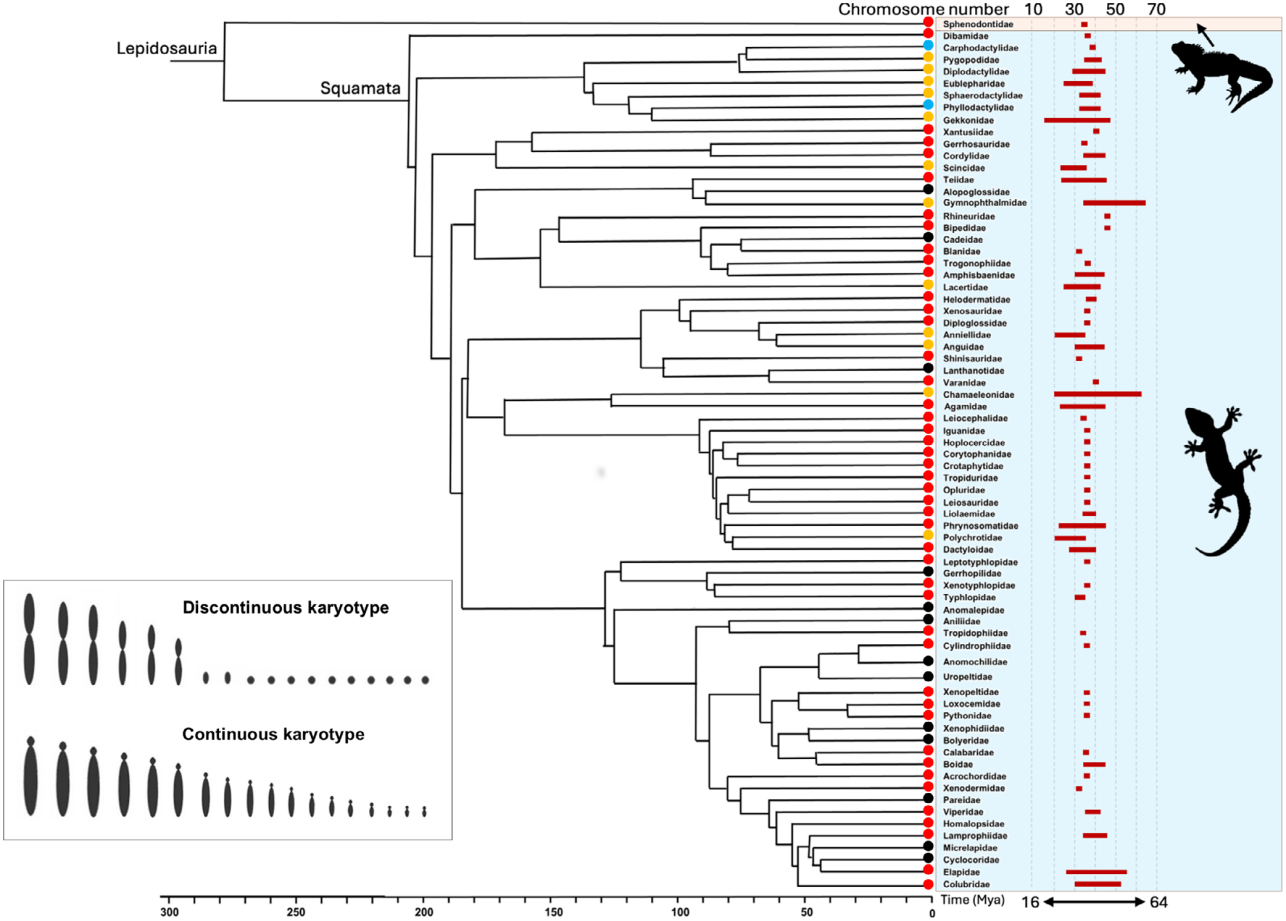
Phylogenetic relationships of Rhynchocephalia and Squamata illustrating the evolution of chromosome number across families. The known range of diploid chromosome numbers is indicated for each family by a red line. Ploidy levels span from diploid to tetraploid, with chromosome counts ranging from 16 to 76 (two‐way arrow in the lower‐right corner). Animal silhouettes represent major lineages (from top to bottom): *Sphenodon punctatus* , *Gekko gecko* . Phylogenetic tree modified from Zheng and Wiens (2016). The inset shows simplified examples of discontinuous and continuous karyotypes similar to those observed in *Brookesia* spp. and *Uroplatus* spp. (Mezzasalma et al. 2021, 2022). Coloured tips on the phylogenetic tree represent the occurrence, within each family, of discontinuous karyotypes (red), continuous karyotypes (sky blue) and both discontinuous and continuous karyotypes (orange). Black tips represent a lack of available information (Mezzasalma et al. 2024).

From a cytogenetic point of view, the extant *Sphenodon* displays a chromosome complement of 2*n* = 36 with 14 macro‐ and four microchromosome pairs and a prevalence of biarmed elements (Norris et al. 2004; O'Meally et al. 2010). Interestingly, *S. punctatus* is characterized by a high GC content and a large genome size of approximately 5 pg per haploid nucleus. This genomic complexity is mostly attributed to the progressive amplification and accumulation of repetitive sequences on multiple chromosome pairs (O'Meally et al. 2010). Overall, the karyotypic features of *S. punctatus* appear intermediate between those typically observed in turtles and squamates, and many of them (such as the low microchromosome number and the large genome size) have been considered derived characteristics, acquired via a progressive accumulation of lineage‐specific macromutations (Norris et al. 2004; O'Meally et al. 2010).

With about 12,000 described species, squamate reptiles represent one of the most diverse and successful groups of extant amniotes. Overall, extant lizards and snakes are currently classified in 43 and 32 families, respectively, showing a global distribution and a remarkable ecological and morphological variability (Uetz et al. 2023; Watanabe et al. 2019).

Despite their extraordinary diversity, lizards and snakes present some generally shared and homogeneous genomic characteristics. In fact, squamate genomes are generally scarcely heterochromatic and with a low DNA content (with an average of 1–3.8 Gbp) when compared to other vertebrates such as mammals, amphibians and non‐squamate reptiles, but similar to values reported for birds (Gregory 2005; Janes et al. 2010). Due to the low heterochromatin content of the squamate genomes, their karyotypes usually lack the distinct banding patterns observed in many other vertebrates. The majority of heterochromatin in squamates is localized on telomeric and centromeric regions as well as on differentiated sex chromosomes (Olmo 2008).

In contrast, the high taxonomic, ecological, morphological and genetic diversity of squamates is reflected at the karyotype level. Over the past decades, squamates have emerged as prominent model systems in the field of evolutionary cytogenetics (see e.g., Deakin et al. 2016; Mezzasalma et al. 2024). This growing research interest stems from the exceptional variability of squamate karyotypes in general structure, total number of chromosomes, the presence of different size classes (macro‐ and microchromosomes), chromosome morphology, genomic content and the distribution of various chromosomal markers (e.g., Mezzasalma et al. 2024; Olmo 2008). Furthermore, squamate reptiles are the only major amniote group characterized by recurrent polyploidization (multiplication of the entire genome complement within an individual). In particular, polyploidy in squamates is mostly present in lizards (in eight families), usually in the form of triploidy (or rarely tetraploidy) associated with hybridization (allotriploidy), obligate or facultative parthenogenesis (asexual mode of reproduction, in which females produce eggs with the same ploidy level as their somatic cells) and the potential of backcrossing, secondary hybridization and reticulate evolution (e.g., Fujita et al. 2020; Mezzasalma, Brunelli, et al. 2023). In some instances, hybrid parthenogenetic lineages may exhibit greater ecological breadth in habitat use compared to their sexual parental species, occupying disturbed, marginal or transitional habitats (Martins et al. 2021; Wright and Lowe 1968).

Overall, the karyotype organization of squamates follows two main general structural patterns, known as discontinuous (or bimodal) and continuous (or unimodal) karyotypes (Figure 2). Discontinuous karyotypes are those showing chromosomes belonging to two clearly distinguishable size classes, macro‐ and microchromosomes. On the contrary, continuous karyotypes lack any clear size/dimensional distinction between macro‐ and microelements showing chromosomes gradually decreasing in length (Mezzasalma et al. 2021, 2024). In vertebrates, the presence of microchromosomes is not exclusive to squamates, as they also occur in cartilaginous and ray‐finned fish, newts and salamanders, turtles, birds and *Sphenodon* (Fornaini et al. 2024; Srikulnath et al. 2021; Stingo and Rocco 2001; Waters et al. 2021). However, squamates are the only amniote group in which discontinuous karyotypes, despite their general prevalence,  frequently alternate with continuous karyotypes among and within families and genera. In particular, among lizard families, 28 show exclusively discontinuous karyotypes, nine show a mixed karyotype structure with both discontinuous and continuous karyotypes, while only Carphodactylidae and Phyllodactylidae show exclusively continuous karyotypes (Figure 2; Mezzasalma et al. 2024). Moreover, discontinuous karyotypes are almost ubiquitous in all the major evolutionary lineages of Iguania, while continuous karyotypes are particularly frequent in Gekkota (Mezzasalma et al. 2024). In turn, the snake karyotype structure appears to be more uniform than in lizards with all the known families displaying only discontinuous karyotypes with the sole exception of Pseudoxyrhophiidae, which also present continuous karyotypes (Mezzasalma et al. 2014, 2024).

From a morphological point of view, it should also be noted that, in squamates, discontinuous karyotypes are often composed of mostly biarmed macrochromosomes, while continuous karyotypes generally include a majority of acrocentric chromosome pairs (Figure 2; see e.g., Deakin and Ezaz 2014; Mezzasalma et al. 2024; Srikulnath et al. 2021).

Concerning diploid chromosome numbers, lizards show karyotypes from 2*n* = 16 (in the ring‐necked clawed gecko *Gonatodes taniae* , Sphaerodactylidae) to 2*n* = 62 (in *Rieppeleon brevicaudatus* and *Notobachia ablephara*, Chamaeleonidae and Gymnophthalmidae, respectively) (Pellegrino et al. 1999; Rovatsos et al. 2017; Schmid et al. 1994). Furthermore, high variability in the total diploid chromosome number can be observed among and within most squamate families and genera, and this variability is generally higher in lizards than in snakes (Figure 2; Mezzasalma et al. 2024; Olmo 2008). Among lizards, 29 families show a karyotype with a variable number of macro‐ and/or microchromosomes, while 16 families display a fixed chromosome formula. In snakes, eight families show a variable chromosome number, while 14 families have a fixed karyotype structure—typically 2*n* = 36 (see Serpentes below) (Mezzasalma et al. 2024).

A few efforts have been made to describe putative ancestral karyotypes in different squamate taxa but considering the high variability in the chromosome number, macro‐ and microchromosomes ratios and chromosome morphology, it appears difficult to provide reliable estimates for most taxa. Nevertheless, molecular cytogenetic data suggest that the ancestral squamate karyotype was likely characterized by ten macrochromosome pairs and several microchromosomes (Deakin et al. 2016). This further implies that a discontinuous karyotype structure should be considered plesiomorphic for the whole group. Furthermore, the ancestral condition in the clade Toxicofera (composed of Anguimorpha, Iguania and Serpentes) likely consisted of seven macrochromosome pairs (four large and three small), which probably originated via chromosome fusions (Deakin et al. 2016).

In Iguania, a karyotype of 2*n* = 36 with six (mostly biarmed) macro‐ and 12 microchromosome pairs is fixed and/or can be considered the ancestral condition in several families such as Iguanidae, Opluridae, Chamaeleonidae, Tropiduridae and Leiosauridae (see e.g., Altmanová et al. 2016; de Matos et al. 2016; Mezzasalma et al. 2024; Mezzasalma, Streicher, et al. 2023). This hypothesis has recently been supported by ancestral karyotype reconstruction and molecular cytogenetics in Chamaeleonidae, where the ancestral condition appears to be fixed in Brookesiinae (the sister clade to all other chameleons), while derived states (with a lower chromosome number) characterize several genera such as *Furcifer*, *Chamaeleo*, *Trioceros* and *Rhampholeon* (Mezzasalma, Streicher, et al. 2023).

In Serpentes, the most common karyotype formula of 2*n* = 36 with eight macro‐ and ten microchromosome pairs is fixed in ten families, and it is generally considered the ancestral chromosome condition of the whole group (e.g., Oguiura et al. 2010; Viana, Ezaz, et al. 2020).

Several alternative evolutionary pathways have been hypothesized to occur in squamates, including a decrease in the chromosome number, an increase in the chromosome number, karyotypic stasis and homoploid hybrid speciation (see e.g., Mezzasalma et al. 2024; Pinto et al. 2023). The first two pathways involve chromosome fusionsand fissions, respectively, as the main types of macromutations shaping karyotype diversification. A decrease in the chromosome number is generally considered the most common evolutionary trend in squamates, but alternative scenarios prevail in different clades and may also coexist in a single clade, as shown, for example, in Gekkota, Chamaeleonidae and snakes (Chrostek et al. 2023; Mezzasalma, Streicher, et al. 2023; Peng et al. 2023). It should also be highlighted that a general decrease in the total chromosome count usually involves the disappearance of microchromosomes via micro‐ to macrochromosome translocations (Mezzasalma et al. 2024; Mezzasalma, Streicher, et al. 2023; Srikulnath, Uno, et al. 2015).

The remarkable karyotypic diversity of squamates provides particularly informative examples of links between chromosome changes and ecological and evolutionary diversification. At the population level, chromosomal races can form contact zones that provide natural systems for examining the effects of karyotypic divergence on gene flow and speciation (Mezzasalma et al. 2024; Rieseberg 2001). A well‐studied example is the *Sceloporus grammicus* complex (Phrynosomatidae), in which chromosomal races differing in multiple rearrangements, including Robertsonian fissions and pericentric inversions, form narrow hybrid zones. In the hybrid zone between the chromosomal races F5 (2*n* = 34) and FM2 (2*n* = 44–46), karyotype is associated with habitat, while chromosomal heterozygosity is associated with reduced fecundity and increased embryo inviability, evidencing how chromosomal differences can interact with ecological differentiation and contribute to reproductive barriers (Reed and Sites 1995; Sites et al. 1995). Similarly, the chromosomal races documented in *Gehyra* (Gekkonidae) further illustrate how chromosomal rearrangements coincide with geographical and ecological divergence, providing informative cytotaxonomic markers of lineage differentiation (Moritz 1986; Oliver et al. 2020).

In general, karyotype changes have been proposed to drive lineage diversification in several lizard and snake groups, including, Chamaeleonidae, Agamidae, Lacertidae, Scincidae, Colubridae, Viperidae, Pseudoxyrhophiidae and Elapidae, primarily through the progressive accumulation of autosomal rearrangements (Deakin and Ezaz 2019; Mezzasalma et al. 2024; Olmo 2008). However, direct evidence of chromosomal divergence as the ultimate cause of reproductive isolation remains limited in most groups, as karyotype changes often occur alongside ecological, genomic and demographic divergence, making the independent contribution of each factor to speciation difficult to disentangle.

Among amniotes, squamate reptiles exhibit the greatest diversity in sex determination and have been regarded as a model system for understanding different evolutionary pathways of genetic and environmental mechanisms. Different evolutionary lineages of squamate reptiles show either environmental sex determination in the form of TSD or genetic sex determination (GSD). Machanisms in species with GSD vary widely and involve simple or multiple sex chromosome systems characterized by either male (XY and X1X2Y) or female (ZW and Z1Z2W) heterogamety (Alam et al. 2018; Mezzasalma et al. 2021). Among lizards, TSD is generally considered the plesiomorphic condition (an observation supported by its presence in *Sphenodon*) and has been previously reported in ten families, but its occurrence in four of them is currently considered debated (Mezzasalma et al. 2021). Conversely, GSD predominates in most lizard families either with male heterogamety (21 families) or female heterogamety (13 families). Sex chromosomes in lizards vary from cryptic, homomorphic pairs to highly differentiated (heteromorphic and heterochromatic) elements. Notably, diverse systems frequently co‐occur within the same lizard families. In particular, when present, TSD always co‐occurs with GSD and both male and female heterogamety are found in several Gekkota and Iguania families (Mezzasalma et al. 2021). Furthermore, GSD with female heterogamety (ZW) was until recently interpreted as occurring in all snakes, but an XX/XY sex chromosome system has been discovered in Scolecophidia and Henophidia (Gamble et al. 2017; Pšenička et al. 2025). These data indicate that transitions among different sex determination systems occurred multiple times during the evolution of squamates involving shifts between TSD and GSD and rapid sex chromosome turnover, potentially representing important mechanisms underlying evolutionary and ecological diversification at different taxonomic levels (Alam et al. 2018; Mezzasalma et al. 2021; Pšenička et al. 2025). A particularly illustrative example is provided by the scincid *Niveoscincus ocellatus*, in which conspecific populations occupying contrasting climatic environments differ in sex‐determination mechanisms. In a highland population, sex is predominantly genetically determined, whereas in a lowland population, sex is influenced by temperature, despite both populations retaining XY‐linked markers (Hill et al. 2018).

### 2.3 Chelonians

Turtles and tortoises constitute a diversified group of terrestrial, marine and freshwater reptiles, which currently includes more than 360 described species, classified into two monophyletic suborders (Cryptodira and Pleurodira) and 14 families (Figure 3; Bista and Valenzuela 2020; Uetz et al. 2023).

**FIGURE 3:**
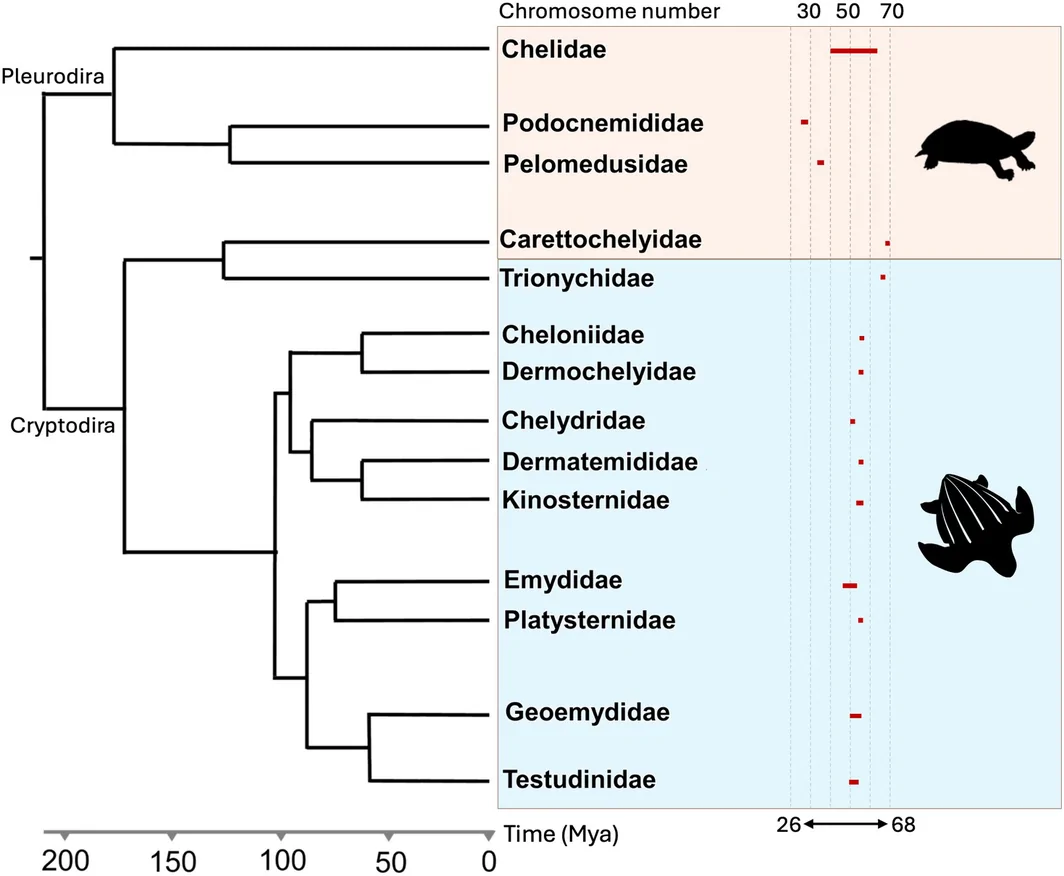
Phylogeny of chelonians illustrating the evolution of diploid chromosome number across families. Major clades are highlighted by colour: Pleurodira (orange) and Cryptodira (turquoise). The known range of diploid chromosome numbers is indicated for each family by a red line. Chromosome numbers range from 26 to 68 (two‐way arrow in the lower‐right corner). Animal silhouettes represent major lineages (from top to bottom): *Foxemys mechinorum, Dermochelys coriacea*. Phylogenetic tree was modified from Bista and Valenzuela (2020).

The modern chelonians have attracted considerable interest in evolutionary cytogenetics. In fact, the group shows a high diversity in chromosome number (ranging from 2*n* = 26 in Podocnemididae to 2*n* = 68 in Carettochelyidae; Figure 3), chromosome morphology and ratio between macro‐ and microchromosome number (Bista and Valenzuela 2020; Clemente et al. 2020, 2021; Valenzuela and Adams 2011).

The observed variability in the karyotypes of chelonians is not uniformly distributed among different evolutionary lineages and while some families display a remarkable karyological diversity, others show a fixed karyotype formula or limited variation in chromosome number and morphology. In particular, the most karyologically diverse groups include Chelidae (2*n* = 40–64), Geoemydidae (2*n* = 50–56) and Emydidae (2*n* = 48–52), while a limited chromosomal variability can also be observed in Testudinidae (2*n* = 50–54), Pelomedusidae (2*n* = 34–36), Podocnemididae (2*n* = 26–28) and Kinosternidae (2*n* = 54–56). In turn, several other families, including Trionychidae (2*n* = 66), Cheloniidae (2*n* = 56), Dermatemydidae (2*n* = 56) and Chelydridae (2*n* = 52) have a fixed karyotype formula and Carettochelyidae (2*n* = 68), Dermochelyidae (2*n* = 56) and Platysternidae (2*n* = 54) represent monospecific taxa (Table S1; Bista and Valenzuela 2020; Clemente et al. 2020, 2021; Uetz et al. 2023; Valenzuela and Adams 2011; Viana, Feldberg, et al. 2020).

So far, true polyploidy has never been observed in chelonians. However, a particular case of mosaicism has been reported in the South American twist‐necked turtle, *Platemys platycephala* (2*n* = 64) (Chelidae), in which a continuum of diploid, triploid and tetraploid mosaicism is linked to geographic variation (Bickham et al. 1985, 1993; Mezzasalma, Brunelli, et al. 2023). Diploid–triploid mosaicism has been documented in Suriname and French Guiana, whereas only diploid individuals have been reported from Bolivia and Brazil, indicating geographic heterogeneity in the occurrence of alternative ploidy states (Bickham et al. 1985, 1993). Triploidy is also associated with males, suggesting that either genome size or ploidy may interact with sex determination. Although the adaptive and evolutionary significance of this unusual condition remains unresolved, *P. platycephala* illustrates how chromosomal variation may occur within species and may interact with geographic structure and reproductive biology, rather than being restricted to divergence among deeply separated lineages.

Chelonian karyotypes generally comprise 8–16 macro‐ and 12–26 microchromosome pairs (Bista and Valenzuela 2020; Clemente et al. 2020, 2021; Valenzuela and Adams 2011). Macrochromosomes are often metacentric or submetacentric, but the number of subtelocentric and acrocentric chromosome pairs varies among families. These chromosome types are generally more prevalent in taxa with higher chromosome numbers, such as representatives of Chelidae and Carettochelyidae (Bickham et al. 1983; Viana et al. 2022).

Apparently, chromosome fusions and fissions constitute the main drivers of karyotype change in turtles. The former are usually associated with the disappearance of microchromosomes via micro‐ to macrochromosome translocations, while the latter generally involve the genesis of relatively small acrocentric pairs starting from larger meta‐ or submetacentric elements (Viana et al. 2022). In different evolutionary lineages, the progressive accumulation of fusion and fission events eventually led to karyotypes with an extreme reduction and increase in the diploid chromosome number, respectively (Viana et al. 2022).

Although generally less common than fusions and fissions, chromosomal inversions and centromere repositioning (affecting both autosomes and sex chromosomes, see below) are also hypothesized to have occurred in several chelonian lineages. These rearrangements contribute to changes in chromosome morphology and may have driven lineage diversification, particularly within the Cheloniidae (Lee et al. 2019; Machado et al. 2020).

It should also be noted that ITRs, which are often related to chromosome rearrangements such as fusions, inversions and translocations (Lin and Yan 2008), occur much rarerely in turtles than in other reptiles, supporting relatively low rates of chromosome evolution despite their diverse chromosome numbers (Clemente et al. 2020).

Recent attempts to reconstruct the ancestral chromosome number in chelonians have proposed 2*n* = 52, while 2*n* = 48 and 2*n* = 56 have been inferred as the plesiomorphic condition for Pleurodira and Cryptodira, respectively (Montiel et al. 2016).

In Pleurodira, a progressive reduction of the chromosome number by chromosome fusion likely occurred in Podocnemididae and Pelomedusidae (with a hypothesized ancestral karyotype of 2*n* = 32 and 2*n* = 36, respectively), while an increase of the chromosome number occurred in Chelidae (ancestral state of 2*n* = 52). Furthermore, Chelidae exhibit divergent tendencies: both further increase (up to 2*n* = 64) and secondary reduction (down to 2*n* = 40) have occurred in different taxa, driving the extensive karyological diversity currently found in the family (Montiel et al. 2016).

In Cryptodira, a karyotype similar to the hypothesized ancestral 2*n* = 56 is conserved in different families, but the most common evolutionary trend is a reduction in the chromosome number by chromosome fusions, up to karyotypes of 2*n* = 50–52. The opposite pathway of progressive increase of the chromosome number by chromosome fissions is mostly observed in Carettochelyidae and Trionychidae (up to 2*n* = 66–68; Montiel et al. 2016).

Chromosomal variation, including changes in chromosome number, morphology and structure, may contribute to lineage differentiation and reproductive isolation in chelonians. The pronounced karyotypic diversity of Chelidae, for example, reflects repeated chromosome fissions and fusions, as well as changes in chromosome morphology and organization (Viana et al. 2022). Such rearrangements may affect meiotic pairing and recombination and potentially contribute to lineage differentiation and reproductive isolation (Rieseberg 2001), although direct evidence for these effects in chelonians remains limited. Conversely, sea turtles (Cheloniidae) generally retain 2*n* = 56, yet interspecific hybridization is well documented, while species show lineage‐specific differences in chromosome morphology and repetitive DNA distribution (Machado et al. 2020). Hybridization is particularly well documented between loggerhead and hawksbill turtles (*Caretta caretta × Eretmochelys imbricata*), with fewer cases involving olive ridleys ( *Lepidochelys olivacea* ) (Vilaça et al. 2023). However, recent studies indicate that adult hybrids are predominantly F1 individuals, often displaying loggerhead‐like migratory behaviour, whereas reduced backcross viability and evidence of hybrid breakdown suggest that postzygotic barriers may emerge in later generations, potentially through cytonuclear interactions (Vilaça et al. 2023).

Sex determination mechanisms in chelonians include TSD and GSD, with the latter featuring either female (ZW) or male (XY) heterogamety (Bista and Valenzuela 2020; Montiel et al. 2016). Overall, TSD occurs in the vast majority of families and species studied and it is considered the most likely ancestral state in chelonians, while male heterogamety has been found in at least four families (Chelidae, Kinosternidae, Geoemydidae and Emydidae) and female heterogamety has been currently documented only in Trionychidae (Bista and Valenzuela 2020; Montiel et al. 2016).

In the whole group, transitions from TSD to GSD and vice versa have been hypothesized to occur from five or six to nine times, often involving the morphological and molecular differentiation of the sex‐specific chromosome (W or Y) via macromutations, heterochromatinization and possibly chromosome degeneration (Bista and Valenzuela 2020; Lee et al. 2019; Montiel et al. 2016).

Overall, the evolutionary and ecological significance of chromosome rearrangements in chelonians is particularly evident from their association with changes in sex‐determining systems and, potentially, reproductive isolation. Comparative analyses indicate that chromosome‐number evolution in turtles is not entirely independent of sex‐determination evolution: rates of chromosome‐number change are approximately 20‐fold higher along phylogenetic branches associated with transitions between TSD and GSD, suggesting that large‐scale chromosomal reorganization and turnover of sex‐determining mechanisms may be evolutionarily coupled (Valenzuela and Adams 2011). Notably, these transitions have been associated with periods of major global temperature change, although a causal role of climate remains unresolved. Thus, chromosome evolution in turtles may reflect evolutionary interactions between chromosomal architecture, sex determination and environmental variation.

## 3 Birds

Birds are the only surviving descendants of the Theropod dinosaurs from which they diverged, and they have diversified into over 11,000 current species over the course of 100 My of evolution (Baron et al. 2017; Gill et al. 2023; Jarvis et al. 2014). Whole‐genome analyses support a division of modern birds into Palaeognathae (Struthioniformes and Tinamiformes) and Neognathae, which includes Galloanseres (Galliformes and Anseriformes) and Neoaves, the largest group, divided into 37 orders (Jarvis et al. 2014).

Avian chromosomes have been the subject of scientific investigation for more than a century, but the current scenario shows that < 10% (1,095) of all described species have been karyotyped and possess a diploid number of chromosomes (Table S2; Degrandi et al. 2020; Guyer 1990). In general, a typical avian karyotype consists of 80 chromosomes organized into two distinct categories clearly distinguishable by size. Macrochromosomes correspond to five to ten pairs varying between 2.5 and 6 μm in length and can be easily classified based on morphology. The remaining chromosomes belong to the category of microchromosomes, which are smaller than 2.5 μm and morphologically indistinguishable (Kretschmer et al. 2018; Rodionov 1996). Historically, microchromosomes were considered unimportant elements of the bird genome; however, it is now known that they are gene‐rich with few transposable elements and exhibit conservation levels comparable to macrochromosomes across bird taxa (Damas et al. 2017; Kretschmer, de Souza, et al. 2021; O'Connor et al. 2019). Their origin has been a subject of debate for decades, and it has been observed that microchromosomes are quite ancient genomic elements, constituting the building blocks of larger chromosomes and they rarely originate from chromosomal fission (Waters et al. 2021). Furthermore, genomic data show that microchromosomes possess significantly higher gene densities and higher rates of recombination compared to macrochromosomes (Hagen et al. 2020), which facilitates more efficient natural selection in these regions.

Birds have a homogametic ZZ sex determination system in males and heterogametic ZW system in females. In Palaeognathae birds, the Z and W chromosomes are similar in size and largely euchromatic. In the common ostrich ( *Struthio camelus* ), the W chromosome exhibits a small pericentromeric heterochromatic region (Nishida‐Umehara et al. 2007). In contrast, Tinamiformes show a more differentiated sex chromosome system, with the Z and W chromosomes differing in size and the W chromosome displaying a substantial accumulation of heterochromatin considered a transitional stage for the highly heterochromatic W found in Neoaves (Garnero et al. 2006; Nishida‐Umehara et al. 2007). Neoaves, in general, have heteromorphic Z and W chromosomes with the W chromosome much smaller than the Z (Kretschmer et al. 2018). Genome sequencing has identified multiple ‘evolutionary strata’ on the avian Z chromosome—regions that stopped recombining with the W at different time points—similar to the evolutionary pattern observed in human XY chromosomes (Zhou et al. 2014). In naturally hybridizing species, this same chromosome shows disproportionately elevated sequence divergence relative to the autosomal background—for example, the Z chromosome of the collared flycatcher ( *Ficedula albicollis* ) and pied flycatcher ( *F. hypoleuca* ) shows markedly higher background divergence and fewer shared polymorphisms than the rest of the genome, indicating that sex chromosomes and autosomes can reach different stages of reproductive isolation during the same speciation event (Ellegren et al. 2012).

However, some species have the enlarged W chromosome due to the accumulation of different repetitive DNA classes (de Oliveira Furo et al. 2017; Gunski et al. 2019; Marshall Graves and Shetty 2001). The Z chromosome has retained a relatively constant size across most avian lineages, typically corresponding to the fourth‐ or fifth‐largest chromosome of the karyotype and preserves a conserved gene content. Despite this conservation, the Z chromosome has experienced extensive intrachromosomal reorganization during avian evolution, exhibiting all possible chromosomal morphologies, including metacentric, submetacentric, acrocentric and telocentric forms. Rather than undergoing frequent interchromosomal exchanges, the Z chromosome has predominantly evolved through inversions, centromere repositioning and other intrachromosomal rearrangements that alter chromosome morphology while largely preserving synteny and gene content (Enguito et al. 2026).

In addition, several examples of multiple sex‐chromosome systems have been described: the Z_1_Z_1_Z_2_Z_2_/Z_1_Z_2_W type in a penguin species, *Pygoscelis adeliae* , where males have 2*n* = 96 (Z_1_Z_1_Z_2_Z_2_) and females 2*n* = 95 (Z_1_Z_2_W; Gunski et al. 2017). A similar system has been reported in boobies (*Sula* spp.) with 2*n* = 76 in males and 2*n* = 75 in females (Pozzobon et al. 2025). Both of these systems, together with a rapidly growing number of other cases across avian diversity, have arisen from sex chromosome–autosome fusions. A fusion mechanism is now recognized as considerably more widespread in birds than previously assumed, despite the group's otherwise conserved karyotype. The best‐documented examples occur within the songbird superfamily Sylvioidea, where whole‐genome comparisons across the majority of the clade's families have confirmed at least five independent fusion events, involving different autosomes fused to the Z and/or W chromosome in different lineages and generating neo‐sex chromosomes composed of a recently fused and an ancestral region (Sigeman et al. 2020, 2022). These neo‐sex chromosomes offer a valuable window into the earliest stages of sex chromosome differentiation, since the added region typically continues recombining for several million years after fusion before recombination suppression spreads across it (Sigeman et al. 2021). Comparable sex chromosome–autosome fusions have also been documented outside Passeriformes, including a fusion of chromosome 11 to the Z and W chromosomes in the common ancestor of parrots (Psittaciformes; see Section 3.3; Huang et al. 2022) and an unusual Z‐autosome Robertsonian translocation in the smooth‐billed ani, *Crotophaga ani* (Cuculiformes; Kretschmer, Gunski, et al. 2020), indicating that this route to sex chromosome enlargement has arisen independently across several distantly related avian lineages rather than being restricted to songbirds.

Approximately 50% of bird species possess a diploid chromosome number ranging between 78 and 82, with 21.63% having the typical avian chromosome number 2*n* = 80 (Degrandi et al. 2020). This conserved chromosome number, along with the stable macrochromosomal structure, has historically supported the concept of chromosomal conservatism in birds. Genomic synteny plots have verified this conservatism, showing that birds have a much lower rate of interchromosomal rearrangements compared to mammals (Zhang et al. 2014). Nevertheless, significant variation exists within the clade, with some species evolving markedly reduced diploid chromosome numbers—such as *Falco columbarius* (Falconiformes) with 2*n* = 40 (Nishida et al. 2008)—and others exhibiting extreme increases, including *Corythaixoides concolor* (Musophagiformes) with 2*n* = 142 (Table S2; Christidis 1990).

Comparative chromosome painting (CCP) has made it possible to establish chromosomal homology between distantly related species, providing a basis for understanding the origin of this wide chromosomal variation. These physical maps have since been cross‐validated by chromosome‐scale genome assemblies, which allow evolutionary breakpoint regions (genomic regions, where rearrangements have frequently occur, sometimes proposed to be hotspot of genomic instability) to be identified at base‐pair level and the rearrangements involved to be inferred (Skinner and Griffin 2012; Zhang et al. 2014). Since 1999, the main advances in this regard have come from macrochromosomal probes from chicken *Gallus gallus* (hereafter GGA1‐9 and GGAZ, following standard chicken chromosome nomenclature) (Griffin et al. 1999). This set of probes has contributed significantly to identifying rearrangements mainly involving macrochromosomes (Degrandi et al. 2017; dos Santos et al. 2019; Kretschmer et al. 2014; Kretschmer, Franz, et al. 2021; Nishida et al. 2008). Probes developed in 2010 from the white hawk *Pseudastur albicollis* (formerly *Leucopternis albicollis* ) enabled the identification of intrachromosomal inversions, revealing that—despite their overall conservation—avian macrochromosomes exhibit notable internal structural variation (de Oliveira et al. 2010; Degrandi et al. 2017; dos Santos et al. 2015; Kretschmer et al. 2014). In 2017, the development of a new set of bacterial artificial chromosome probes enabled comparative analysis of microchromosome homology between *G. gallus* and other avian species (Damas et al. 2017; de Souza et al. 2022; Kretschmer, de Souza, et al. 2021; O'Connor et al. 2019; Ribas et al. 2021). These molecular tools, combined with whole‐genome alignments, have shown that interchromosomal synteny is largely conserved, whereas intrachromosomal inversions are much more frequent than previously suspected, often contributing to local adaptation and speciation (Zhang et al. 2014).

Integrating classical and molecular cytogenetics with chromosome‐level assemblies led to the proposal of putative ancestral karyotype (PAK) of birds, with 2*n* = 80 and a macrochromosomal organization similar to that of *G. gallus* (Figure 4; Griffin et al. 2007). Genomic reconstructions have further refined the avian PAK, identifying specific gene orders within the microchromosomes that characterize the ancestral avian state (Damas et al. 2018; O'Connor et al. 2018). The primary exception is chromosome GGA4, which in the ancestral state corresponded to two distinct chromosomes—PAK4 and PAK10 (Griffin et al. 2007). In the following sections, we examine key events in karyotype evolution by analysing diploid number variation and chromosome homology maps, contextualised within the framework of the most up‐to‐date avian phylogeny (see Figure 4).

**FIGURE 4:**
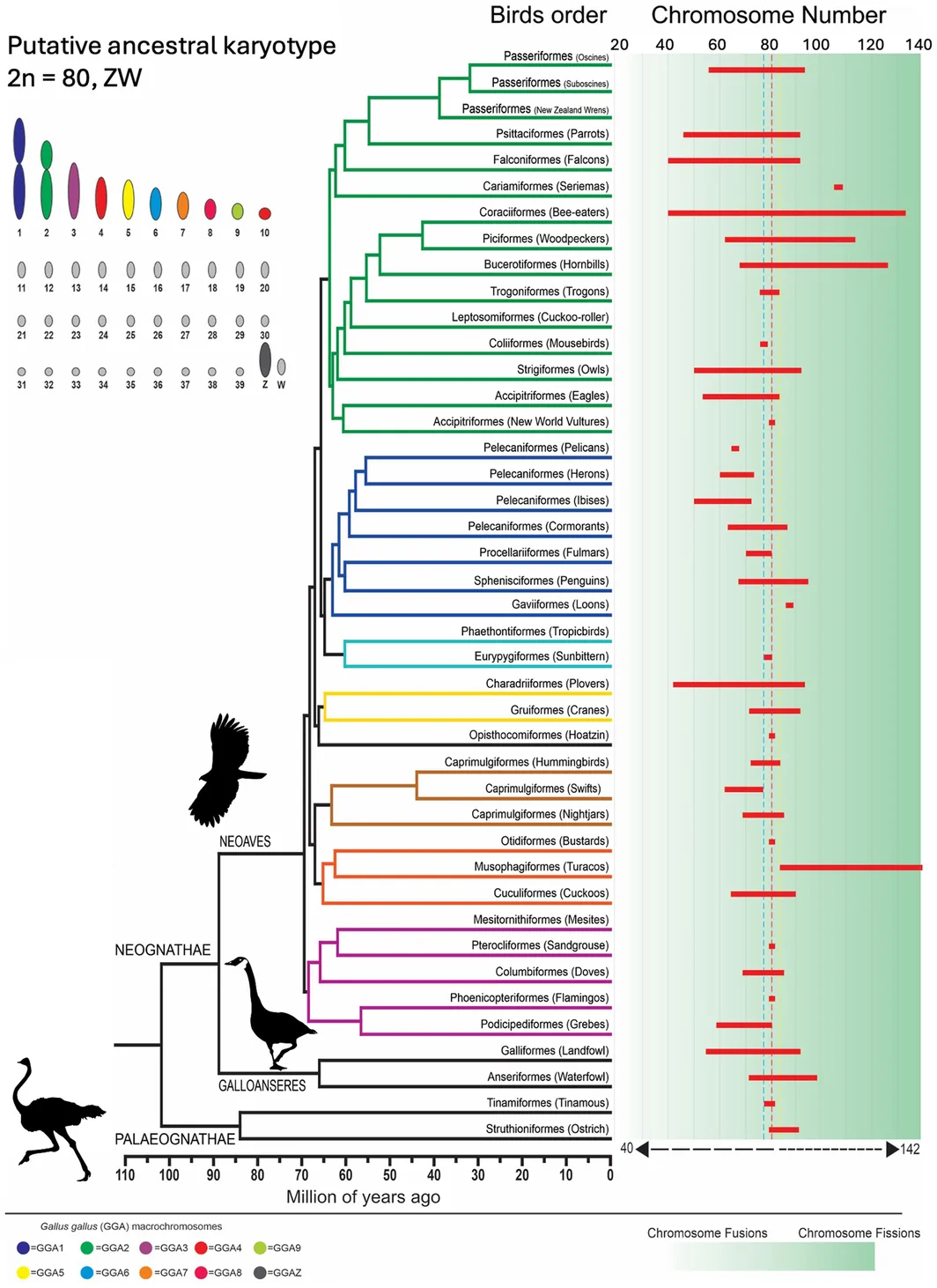
Avian phylogeny depicting chromosome number variation across all orders. Red bars represent the range of described diploid chromosome numbers for each order. Chromosome numbers range from 40 to 142 (two‐way arrow in the lower‐right corner). The chromosome ideogram in the top left illustrates the putative ancestral karyotype (2*n* = 80; Griffin et al. 2007) with the legend of chromosome painting probes homologous to *Gallus gallus* chromosomes (bottom left). Order‐specific interchromosomal rearrangements are indicated along the branches, with fissions shown in red and fusions in blue. Phylogenetic relationships follow Jarvis et al. (2014), and chromosome number data are based on the Bird Chromosome Database (Degrandi et al. 2020). Animal silhouettes represent Neoaves (*Ictinaetus malaiensis*, top), Galloanseres (*Branta canadensis*, middle) and Palaeognathae ( *Struthio camelus* , bottom).

Because avian karyotypes are conserved in chromosome number, diploid number itself is not a comparatively valuable cytotaxonomic marker in birds relative to the reptiles and mammals discussed elsewhere in this review. Nevertheless, chromosomal variation still bears on avian ecology and evolution in several ways. Birds accumulate postzygotic reproductive incompatibilities at a markedly lower rate than mammals, a pattern that has been linked to their generally stable karyotype (Ellegren 2010). Consistent with this, several avian orders (e.g., the waterfowl, Anseriformes) hybridize extensively in the wild despite differences in chromosome number among the interbreeding species, indicating that karyotypic divergence alone rarely constitutes a strong barrier to gene flow in this class (Ottenburghs 2018). Where large‐scale karyotypic reorganization occurs, it tends to cluster around major radiation events (see Section 3.3), implying a link between bursts of chromosomal turnover and ecological diversification rather than gradual evolutionary change (Suh et al. 2015).

### 3.1 Palaeognathae 2*n* (78–92)

Palaeognathae includes ostriches, rheas, kiwis, cassowaries, emus and tinamous. Chromosome numbers have been determined for 12 species of this group and range from 2*n* = 78 to 92 (Figure 4; Table S2). Most species have a typical karyotype with 2n = 78 or 80 chromosomes with the exception of *Casuarius casuarius* (2*n* = 92). CCP studies using GGA1‐9 and GGAZ probes evidenced that each probe hybridized to a single pair of chromosomes in all six species analysed, with the exception of GGA4, which hybridized to both the fourth‐largest chromosome and an additional pair of microchromosomes (Nishida‐Umehara et al. 2007). This pattern corresponds to the ancestral PAK4/PAK10 condition (Griffin et al. 2007), indicating that palaeognaths retain the ancestral state of this chromosome. These observations also confirm high homology among palaeognath karyotypes and that an increase in the number of *C. casuarius* chromosomes to 92 may be attributed to fissions of chromosomes smaller than chromosome 9 (ei., those not painted by the GGA1‐9 and GGAZ probes). However, further studies are necessary to test this hypothesis (Nishida‐Umehara et al. 2007).

Direct evidence linking chromosome variation to speciation or hybridization is limited in Palaeognathae. Deep divergence and a small number of extant lineages in this group leave few opportunities to observe natural hybridization or intraspecific chromosomal races, and the CCP‐based homology described above has so far been used mainly to reconstruct ancestral synteny rather than to test speciation models directly.

### 3.2 Galloanseres 2*n* (54–98)

The Galloanseres is a monophyletic group that includes the orders Galliformes (megapodes, chachalacas, curassows, guans, guinea fowl, New World quail, pheasants and allies) and Anseriformes (screamers, magpie goose, ducks, geese and swans). Within Galliformes, diploid numbers vary considerably, spanning from 54 to 92 chromosomes (Figure 4; Table S2). CCP studies have been carried out on 16 species from 13 different genera (Guttenbach et al. 2003; Kasai et al. 2003; Shibusawa, Nishibori, et al. 2004; Shibusawa, Nishida‐Umehara, et al. 2004). Nine of them have karyotypes resembling the PAK. The fusion of PAK4 and PAK10 occurred in six species, as seen in GGA4 in chicken (Guttenbach et al. 2003; Kasai et al. 2003; Shibusawa, Nishida‐Umehara, et al. 2004). Other chromosomal rearrangements identified, include a centric fission of GGA2 and fusions of GGA4/GGA9, GGA8/GGA9, GGA6/GGA7 and GGA6/GGA8 (Guttenbach et al. 2003; Shibusawa, Nishibori, et al. 2004). Diploid numbers in Anseriformes range from 74 to 98 chromosomes (Figure 4; Table S2). CCP showed that all species have karyotypes similar to the PAK; however chromosomal fissions are inferred to have occured in species with chromosome numbers higher than 80 (Rodrigues et al. 2014).

Galloanseres provides some of the best‐documented examples of extensive interspecific hybridization among vertebrates, despite differences in chromosome number and organization between species. Hybridization is pervasive among waterfowl (Anatidae), with the Mallard ( *Anas platyrhynchos* ) alone documented to hybridize with many other duck and goose species. Similarly extensive hybridization network is found among gamebirds (Galliformes), where it centers on the Common Pheasant ( *Phasianus colchicus* ) (Ottenburghs 2018). Because chromosome number varies considerably within both Anseriformes (2*n* = 74–98) and Galliformes (2*n* = 54–92), these hybridization events routinely occur between karyotypically divergent parents, yet postzygotic isolation in this clade appears to depend more on the specific rearrangements and ecological and behavioural barriers than on the raw magnitude of chromosome‐number difference (Ottenburghs 2018)—a pattern broadly consistent with the limited postzygotic effect of karyotypic divergence documented in the rock‐wallabies of the genus *Petrogale* (Section 4.2) and in *Crocodylus* (Section 2.1). This makes Anseriformes and Galliformes valuable systems for disentangling the relative contributions of chromosomal, genic, and ecological factors to reproductive isolation.

### 3.3 Neoaves 2*n* (40–142)

Neoaves includes 37 orders with a wide diversity of species, including penguins, parrots, falcons, eagles, kingfishers, owls and others. It is the bird group that harbours the greatest variation in chromosome number, ranging from 2*n* = 40 to 142 (Figure 4; Table S2; Degrandi et al. 2020). Comparative genomic analysis across the Neoaves phylogeny suggests that large‐scale karyotypic explosions (rapid bursts of fusions and fissions) often coincide with major radiation events following the Cretaceous‐Paleogene boundary (Suh et al. 2015). This temporal clustering suggests that bursts of chromosomal reorganization may have been linked to the rapid ecological and niche diversification of early Neoaves. Within Charadriiformes (2*n* = 42–94), *Burhinus oedicnemus* (2*n* = 42) stands out because of extensive fusions involving both macro‐ and microchromosomes (Nie et al. 2009). In Falconiformes (2*n* = 40–92), CCP and genomic mapping show that the reduction of 2*n* from 52 to 40 in *Falco* was due to multiple fusions among macrochromosomes, as well as between macro‐ and microchromosomes (Joseph et al. 2018; Nishida et al. 2008). In Accipitriformes (eagles, hawks, vultures and allies, 2*n* = 54–82), many species of the Accipitridae family have 2*n* = 66 chromosomes (Degrandi et al. 2020). CCP has demonstrated that the net reduction in the chromosome number from the ancestral state occurred through a dramatic karyotype reorganization, with multiple fissions of macrochromosomes and subsequent fusion events between the resulting fragments and microchromosomes (Carvalho et al. 2021; de Oliveira et al. 2005, 2013). In Psittaciformes, the order that includes parrots and cockatoos, the diploid numbers range between 46 and 92 chromosomes (Figure 4; Table S2). *Myiopsitta monachus* (Psittacidae; 2*n* = 48), exhibits extensive chromosomal rearrangements as revealed by CCP, including numerous fissions in macrochromosomes and multiple fusions involving micro‐ and macrochromosomes (Furo et al. 2020).

In contrast to these fusion‐driven reductions, within the order Piciformes (2*n* = 62–114), *Ramphastos tucanus tucanus* and *Pteroglossus inscriptus* both exhibit a notably high diploid number of 112 chromosomes. CCP revealed extensive fissions of GGA1‐GGA5, resulting in the formation of microchromosomes, while GGA6‐10 each hybridized to a single chromosomal pair (Kretschmer, dde Oliveira Furo, et al. 2020). The order Coraciiformes, which includes rollers, kingfishers and bee‐eaters, also shows a wide chromosome number variation (2*n* = 40–132) and highly heterogeneous karyotypes (Degrandi et al. 2018, 2020). In the kingfishers, *Chloroceryle americana* (2*n* = 84) and *Megaceryle torquata* (2*n* = 94), the difference of ten chromosomes suggests five fissions of macrochromosomes (Degrandi et al. 2018). This inference is based on diploid numbers and classical karyotypes, because CCP has not yet been conducted in any species of Coraciiformes. Passeriformes (2*n* = 56–94) is the largest avian order, comprising more than 6700 species (Gill et al. 2023). To date, CCP has been applied to only a small fraction of them and has mostly revealed conserved karyotypes. A fission of GGA1 is a recurring feature among Passeriformes. The exception is *Tolmomyias sulphurescens* (Rhynchocyclidae), which has a reduced number of 2*n* = 60 compared with the typical passerine karyotype of about 2*n* = 80 (Degrandi et al. 2020). Its karyotype reveals extensive chromosomal fusion events among macro‐ and microchromosomes, including GGA4q/GGA3, GGA6/GGA12/GGA14, GGA7/GGA8, GGA9/GGA10, GGA4p/GGA11, GGA18/GGA15, GGA13/microchromosome, GGA28/microchromosome. In addition, GGA1 is not fissioned in *T. sulphurescens* , which most likely reflects a secondary fusion of GGA1p and GGA1q that restored the ancestral unfissioned state, rather than retention of that state (Kretschmer, Franz, et al. 2021).

Because these karyotypic explosions are concentrated in specific Neoaves lineages rather than distributed evenly across the clade, chromosome number remains a useful cytotaxonomic marker mailnly within groups such as Falconiformes, Accipitriformes and Coraciiformes, where diploid chromosome number diverges sharply between closely related genera (Carvalho et al. 2021; Degrandi et al. 2020; Joseph et al. 2018) even though it stays essentially invariant across most of the avian tree (Degrandi et al. 2020). Because the same diploid number can arise through different rearrangements, it is most reliable when combined with homology data from chromosome painting or genome comparisons (Kretschmer, Franz, et al. 2021).

## 4 Mammals

Mammals, the descendants of synapsid ancestors, have diversified into more than 6000 living species (6815 according to the Mammal Diversity Database v. 2.2 by the American Society of Mammalogists; Burgin et al. 2018). Modern phylogenetics and phylogenomics support diversification of modern mammals into six primary clades: Monotremata, Marsupialia, Afrotheria, Xenarthra, Laurasiatheria and Euarchontoglires (Asher 2018; Bininda‐Emonds et al. 2007; Murphy et al. 2001). Afrotheria, Xenarthra, Laurasiatheria and Euarchontoglires form the Eutheria, which, together with Marsupialia, constitute the therian mammals (Figure 5).

Over the past century of cytogenetic research in mammals, a complex landscape of chromosomal organization and variation in chromosome number has been observed. However, to date, cytogenetic analysis has been developed for only a limited number of species, and therefore, more research is required in this field. The largest available database, the Animal Chromosome Count (ACC) database release 1.0.1 (Román‐Palacios et al. 2021), contains only 1327 records for mammals, covering approximately 20% of decsribed mammal species.

CCP and other types of in situ hybridization have helped in understanding the evolutionary relationships and divergence of mammals, illustrating the dynamic nature of mammalian chromosomes (Ferguson‐Smith and Trifonov 2007). These studies highlighted frequent chromosomal fissions and fusions as adaptive mechanisms that have shaped mammalian genomes throughout their evolutionary history (Murphy et al. 2005). Modern advances in computational genomics have made it possible to compare genomes down to the nucleotide level, identify macro‐ and microsyntenic regions (Yu et al. 2024) and identify and characterize regions of evolutionary chromosome breakpoints (Damas et al. 2022; Farré et al. 2019).

Cytogenetic studies have demonstrated that all mammalian species examined are diploid (Table S3). Previously, tetraploidy was assumed for the plains viscacha rat ( *Tympanoctomys barrerae* ) based on its high diploid number (2*n* = 102) and a genome size double that of its closest relatives (Gallardo et al. 1999). However, chromosome painting (Svartman et al. 2005), whole‐genome sequencing and transcriptome sequencing (Evans et al. 2017) subsequently confirmed that its genome is diploid.

A typical mammalian karyotype contains 36–60 chromosomes (Table S3). Furthermore, over 85 mammalian species are known to carry B chromosomes, the functions of which remain unclear (Vujošević et al. 2018). These B chromosomes are additional to the standard set and their numbers vary significantly across different species—not just among mammals, but across a broad range of taxa. Mammalian genomes represent a mosaic of isochores—genomic segments with a characteristic GC and dispersed repetitive DNA content (Bernardi 1993). This heterogeneity enabled the development of differential chromosome staining techniques and provided a possibility to compare homologous regions from different mammalian taxa (Graphodatsky et al. 2011).

A comprehensive synteny analysis based on chromosome‐scale scaffolded genomes has enabled the reconstruction of ancestral karyotypes for various mammalian clades, mostly confirming and refining CCP data and even revealing the conservation of some synteny blocks shared with the common amniote ancestor for over 300 My (Damas et al. 2022).

Beyond documenting this diversity, chromosomal variation in mammals has direct consequences for reproductive isolation and lineage divergence. Differences in chromosome number or structure between diverging populations can act as postzygotic barriers to gene flow, and karyotype is routinely used as a cytotaxonomic marker to delimit morphologically cryptic species. In addition, where species or races with different chromosome numbers occur, the outcome of hybridization provides a direct test of how much karyotypic divergence a genome can tolerate before reproductive isolation is complete. Representative examples of each of these phenomena are discussed within the relevant clades below.

Mammalian sex chromosomes, particularly the X and Y chromosomes in therian mammals, are among the oldest and most conserved, with significant differentiation and specialization. These chromosomes evolved approximately 150 Mya (Veyrunes et al. 2008). The X chromosome, one of the most conserved elements of the therian karyotype, has retained many ancestral genes, whereas the Y chromosome has largely degenerated, preserving only a limited set of essential genes. This degeneration did not proceed uniformly along the chromosome: it occurred in a series of ‘evolutionary strata’—physically discrete regions that ceased recombining between the sex chromosomes at successive points in time and consequently show different degrees of X‐Y sequence divergence. In humans, at least four such strata have been identified, ranging from the oldest, dating to shortly after the mammal‐bird split around 240–320 Mya, to the youngest, which formed much more recently within the primate lineage (Lahn and Page 1999). This finding directly parallels the same stepwise strata pattern already described for the avian Z chromosome (Section 3). However, some rodent species exhibit unusual sex‐determining systems, such as fertile XY females and the complete loss of the Y chromosome (see review Saunders and Veyrunes 2021). These systems provide unique natural models to study the fundamental processes of sex determination in mammals.

### 4.1 Monotremata

Monotremata, or monotremes, encompass five extant species: semi‐aquatic platypus (family Ornithorhynchidae) and four terrestrial echidnas (Tachyglossidae). These mammals exhibit a combination of reptilian and mammalian traits such as egg‐laying, fur and lactation (Warren et al. 2008). Monotremes are the only extant mammalian outgroup to therians, from which they diverged 187 Mya (Zhou et al. 2021). Consequently, analysing chromosome evolution in monotremes is crucial for understanding early mammalian genome evolution and the divergence of sex chromosome systems.

Monotreme karyotypes were obtained for both families; diploid chromosome numbers range from 52 in platypus ( *Ornithorhynchus anatinus* ) to 64 in the female echidnas (Table S3 and citations therein). Monotremes have unique karyotypes that primarily feature metacentric, submetacentric or subtelocentric chromosomes, with no acrocentric chromosomes observed. Platypus has 26 chromosome pairs (2*n* = 52; van Brink 1959), including a complex sex chromosome system with five X and five Y chromosomes that form a multivalent chain during meiosis (Casey et al. 2017; Rens et al. 2007). This sex chromosome system likely evolved through multiple reciprocal translocations, leading to an ancestral ring structure with several pseudoautosomal regions (PARs) that promote recombination (Zhou et al. 2021). The platypus karyotype includes small chromosomes that are difficult to distinguish, resembling reptilian microchromosomes in general architecture, but larger in size than those typically found in birds and non‐avian reptiles (Zhou et al. 2021). The short‐beaked echidna ( *Tachyglossus aculeatus* ) possesses a slightly different yet more conserved karyotype, with males having 63 and females 64 chromosomes (Bick and Jackson 1967; Zhou et al. 2025).

Comparative studies of platypus and echidna chromosomes have provided significant insights into monotreme chromosomal evolution. Recent genomic studies suggest that the ancestral karyotype of extant monotremes consisted of 64 elements (Zhou et al. 2025). Monotreme sex chromosomes originated from a different linkage group than those of eutherians. They arose through a series of sex chromosome‐autosome translocations in the common ancestor of extant monotremes, followed by lineage‐specific additions of ancestral autosomes into the sex chromosome chain: X4 in the platypus and X5 in the echidna (Zhou et al. 2025).

The reconstruction of the monotreme ancestral karyotype has challenged the previously proposed putative mammalian karyotype (2*n* = 40; Damas et al. 2022) and instead suggests an ancestral karyotype consisting of 30 or 32 chromosome pairs (2*n* = 60; Zhou et al. 2025 or 2*n* = 64; Liu and Zhou 2025).

With only five extant species and no documented interspecific hybrids or intraspecific chromosomal races, monotremes offer little direct evidence linking karyotype variation to speciation or gene flow.

### 4.2 Marsupialia

Marsupials, a diverse group of over 300 species found in the Americas and Australasia, are characterized by a distinctive reproductive strategy in which offspring are born at an early stage of development and continue to mature within a maternal pouch. This unique characteristic sets them apart from eutherian mammals, from which they diverged approximately 160 Mya (Figure 5; Kumar et al. 2022). Marsupials have distinctive karyotypes with a typically small number of large chromosomes. Their diploid chromosome numbers range from 10 in the female swamp wallaby ( *Wallabia bicolor* ) to 32 in the rufous bettong ( *Aepyprymnus rufescens* ) (Román‐Palacios et al. 2021). The Dasyuridae family exhibits extreme karyotypic conservation, with most species having a 2*n* = 14 karyotype, thought to resemble the ancestral marsupial karyotype (Westerman et al. 2010). In contrast, the Macropodidae family (kangaroos and wallabies) shows significant chromosomal rearrangements, with chromosome numbers from 2*n* = 10 to 2*n* = 24. The ancestral macropod karyotype is believed to have had 2*n* = 22 chromosomes, evolving to current karyotypes through fissions and fusions (Deakin 2018).

**FIGURE 5:**
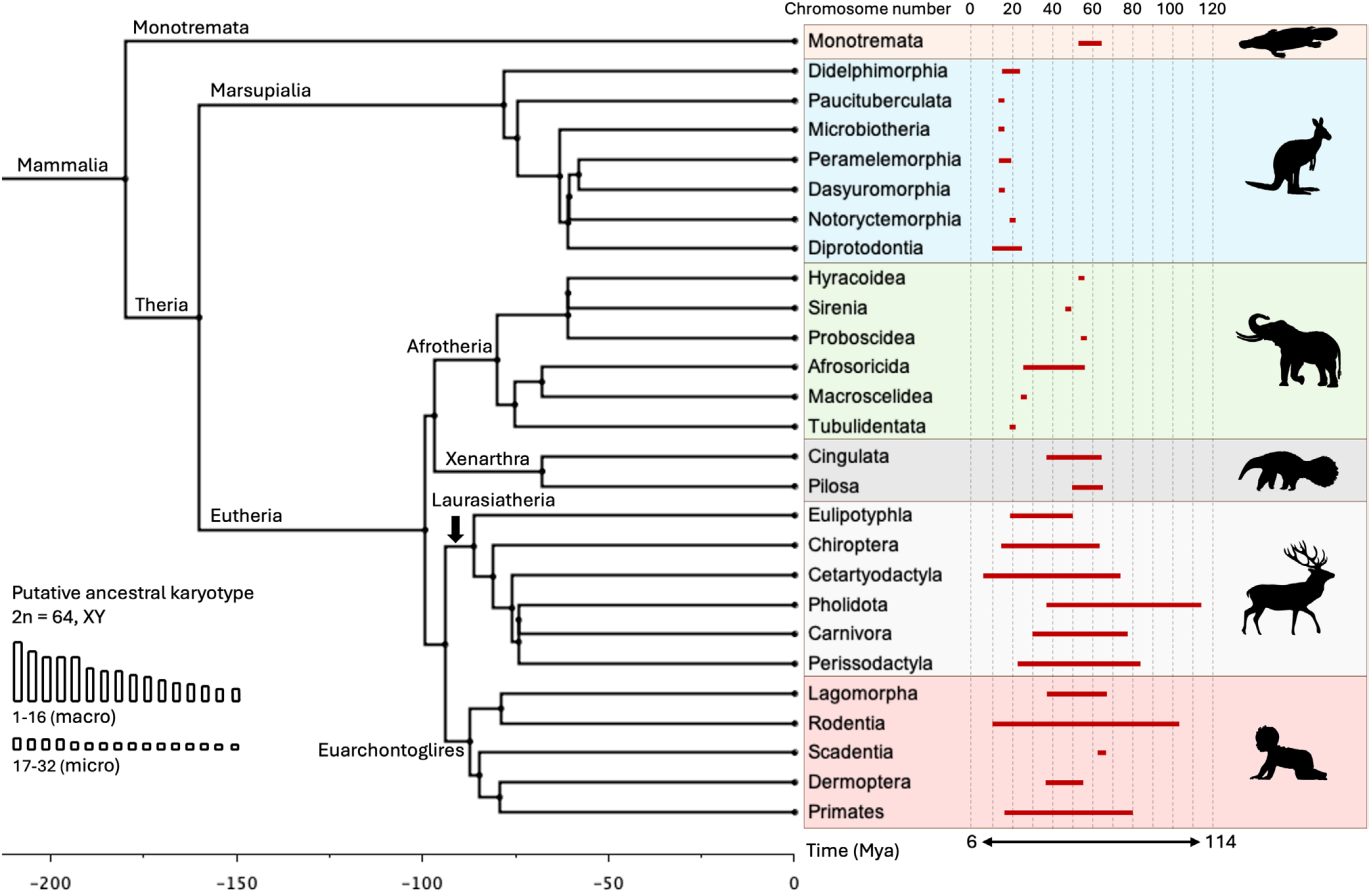
Mammalian phylogeny depicting chromosome number variation throughout orders. Monotremata, Marsupialia, Afrotheria, Xenarthra, Laurasiatheria and Euarchontoglires are highlighted with orange, turquoise, green, light grey, pearl and pink colours, respectively. The range of diploid chromosome numbers is indicated for each order by a red line. The overall range of diploid chromosome numbers, from 6 to 114, is shown by a two‐way arrow in the lower‐right corner. Putative ancestral karyotype shows 2*n* = 64 chromosomes containing 16 macro‐ and 16 microchromosome pairs with XX/XY sex chromosome system present in all mammalian lineages examined. Animal silhouettes are ordered from top to bottom: *Ornithorhynchus anatinus* , *Osphranter rufus*, *Loxodonta africana* , *Cervus elaphus* and *Homo sapiens* . Phylogenetic tree was generated using Timetree of Life (TimeTree.org; Kumar et al. 2022) and visualized in FigTree, v.1.4.4.

Marsupials have experienced considerable chromosomal reorganization. For example, the swamp wallaby exhibits a unique sex chromosome system with an X‐autosome fusion, resulting in XX females and XY_1_Y_2_ males (Toder et al. 1997). Chromosome painting (de Leo et al. 1999; Glas et al. 1999; Rens et al. 2001; Rens, O'Brien, Fairclough, et al. 2003) and gene mapping (Deakin et al. 2013) have revealed conserved segments across various marsupial species. While dasyurids display high karyotypic stasis, macropods exhibit extensive chromosomal reshuffling, providing insights into the role of these rearrangements in speciation and genome evolution.

The ancestral marsupial karyotype likely had fewer chromosomes than modern macropods. The ancestral marsupial karyotype comprises 14 chromosomes, featuring 18 conserved autosomal blocks and one X chromosome block (Rens, O'Brien, Fairclough, et al. 2003; Rens, O'Brien, Graves, et al. 2003). Chromosome painting techniques have confirmed the high degree of evolutionary stability of these conserved chromosomal elements across various marsupial species.

A particularly informative example of chromosomal speciation in marsupials comes from a group not otherwise discussed above: the rock‐wallabies (genus *Petrogale*, Macropodidae). Its 17 species occupy often isolated rocky‐outcrop habitats and comprise 23 distinct karyotypes distinguished mainly by Robertsonian fusions—the greatest chromosomal diversity documented in any marsupial genus (Eldridge and Close 1993; Potter et al. 2022). This diversity arose during a recent radiation (~0.4–1.6 Mya); the small effective population sizes typical of these isolated colonies are thought to have facilitated the fixation of underdominant rearrangements by genetic drift, consistent with the canalization model discussed in Section 6. Karyotype remains the most reliable cytotaxonomic character for distinguishing *Petrogale* species, several of which are otherwise morphologically indistinguishable (Eldridge and Close 1993). Genomic data have since complicated the simple chromosomal‐speciation narrative for this genus. However, species carrying complex, monobrachially homologous Robertsonian fusions nonetheless show substantial admixture and introgression across karyotypic boundaries, indicating that chromosome rearrangement alone does not fully explain reproductive isolation and that genic divergence should also be considered (Potter et al. 2022)—a limited postzygotic effect of karyotypic divergence that parallels the pattern documented within *Crocodylus* (Section 2.1).

### 4.3 Afrotheria

Afrotheria is a distinctive clade of placental mammals comprising six African‐origin orders: Proboscidea (elephants), Sirenia (manatees and dugongs), Hyracoidea (hyraxes), Macroscelidea (elephant shrews), Afrosoricida (golden moles and tenrecs) and Tubulidentata (aardvarks). These species display significant morphological diversity and occupy various ecological niches, attributed to their prolonged evolutionary history and isolation in Africa (Hedges 2001).

Afrotherian species are characterized by relatively large genome sizes (Kasai et al. 2013) and exhibit unique karyotypes with varied chromosomal structures. For example, the aardvark ( *Orycteropus afer* ) has a 2*n* = 20 karyotype, while the African elephant ( *Loxodonta africana* ) has 2*n* = 56 (Román‐Palacios et al. 2021). Comparative chromosome painting between humans and various Afrotherian species, including the *O. afer* , *L. africana* and golden mole ( *Chrysochloris asiatica* ), has revealed important aspects of their chromosomal structure. The aardvark's karyotype is highly conserved and suggests a mammalian ancestral karyotype with 2*n* = 44, featuring nine chromosomes with complete conserved synteny with humans, such as human chromosomes 5–6 (HSA5–6, using standard human‐chromosome‐based comparative nomenclature), HSA9 and HSA11. Other chromosomes retain syntenic blocks but are rearranged in the ancestor, like HSA 3/21 and HSA 16/19q syntenies across Afrotherian species (Yang et al. 2003). Unique syntenic associations like HSA 3/21/5 in the aardvark, golden mole and elephant‐shrew ( *Elephantulus edwardii* ) highlight a distinctive chromosomal signature of Afrotheria (Robinson et al. 2004). The identification of shared chromosomal breakpoints at fragile sites in these species emphasizes the non‐random nature of chromosomal evolution and identifies regions susceptible to evolutionary reorganization. In aardvarks, fragile sites like HSA1p11, HSA1q18 and HSA2q13 align with evolutionary breakpoints, suggesting that these regions are hotspots for chromosomal rearrangement. Comparative analysis in the golden mole and elephant shrew also shows a correlation between fragile sites and evolutionary breakpoint regions, with conserved sites like HSA1q32 in the golden mole and HSA3q24 in the elephant shrew being particularly significant (Ruiz‐Herrera and Robinson 2007).

Direct evidence linking chromosomal variation to speciation or hybridization remains sparse for Afrotheria: no natural hybridization between species with divergent karyotypes has been documented, and CCP data have so far been used mainly to reconstruct ancestral synteny and breakpoint hotspots rather than to test speciation models directly.

### 4.4 Xenarthra

Xenarthra, which includes sloths, armadillos and anteaters, is distinguished by the presence of xenarthrous vertebrae, additional articulations between the dorsal and lumbar vertebrae and represents a unique clade in the mammalian phylogenetic tree. The study of chromosome evolution in Xenarthra is crucial as it provides insights into the early stages of mammalian genome evolution and the diversification of sex chromosome systems.

Xenarthrans exhibit distinctive karyotypes. The chromosome numbers in this group range widely, from 2*n* = 38 in the armadillo ( *Tolypeutes matacus* ; Jorge et al. 1977) to 2*n* = 65 in the sloth ( *Choloepus didactylus* ; Dobigny et al. 2005). CCP with human chromosome‐specific probes, has been applied to several xenarthran species, providing valuable comparative data on their karyotype evolution. For instance, *Bradypus torquatus* (2*n* = 50) and *B. variegatus* (2*n* = 54) have been studied, revealing in toto conservation of 11 human syntenic blocks in both species (Azevedo et al. 2012).

Based on comparative analyses, a PAK of Xenarthra with 48 chromosomes has been proposed (Azevedo et al. 2012). This ancestral karyotype is distinguished from the ancestral eutherian karyotype by the presence of the HSA 7/10 association and the division of HSA8 into three blocks, rather than the two blocks found in the eutherian ancestor. Notably, the ancestral eutherian association HSA 16/19 was not detected in *Bradypus* species (Azevedo et al. 2012), highlighting the unique evolutionary path of this group. These chromosomal features support the monophyly of Xenarthra, making it the second placental mammal superorder, after Afrotheria, to have a chromosomal signature indicative of its monophyly.

The formation of the unique chromosomal rearrangements observed in Xenarthra has significantly influenced the evolutionary trajectory of this group. For instance, the karyotype of *C. hoffmanni* (2*n* = 50) closely resembles the proposed ancestral eutherian karyotype, reinforcing the hypothesis of a 2*n* = 48 ancestral eutherian complement (Svartman et al. 2006). This resemblance suggests that Xenarthra may hold a basal position in the eutherian phylogenetic tree.

Similarly, ecological and speciation‐related data remain limited for Xenarthra. The karyotypic difference between the two *Bradypus* species discussed above (2*n* = 50 vs. 2*n* = 54) raises the possibility of chromosomally mediated reproductive isolation, but natural hybridization has not been systematically investigated in this group.

### 4.5 Laurasiatheria

Laurasiatheria, a diverse group of placental mammals, encompasses several well‐known orders such as Carnivora (dogs, cats and relatives), Perissodactyla (horses, tapirs, rhinos), Chiroptera (bats) and Cetartiodactyla (cattle, pigs, camels, hippopotamuses and whales). Across Laurasiatheria as a whole, chromosome numbers span from 2*n* = 6 in the Indian muntjac ( *Muntiacus muntjak* ; Wurster and Benirschke 1970) to 2*n* = 114 in the white‐bellied pangolin (*Phataginus tricuspis*; Houck et al. 2023), reflecting a broad karyotypic range that spans that of all mammals (Figure 5, Table S3). The study of chromosomal evolution in Laurasiatheria offers significant insights into the mechanisms of karyotype evolution, speciation and genomic adaptations that have facilitated these mammals' adaptation to various ecological niches.

Carnivora, a major order within Laurasiatheria, displays considerable karyotypic diversity. CCP studies have identified conserved syntenic segments across different families, although some species exhibit highly rearranged karyotypes. Diploid chromosome numbers vary considerably in the Carnivora. The American mink ( *Neovison vison* ) has the smallest number of chromosomes (2*n* = 30) (Makino 2011). Representatives of the genus *Canis* have 2*n* = 78 (O'Brien et al. 2005). The PAK of Carnivora is characterized by a chromosome number of 2*n* = 38 (Nash et al. 2008) or 42 (Nie et al. 2012). Significant rearrangements are evident in families such as Canidae (dogs) and Ursidae (bears), where numerous fissions and fusions have reshaped their karyotypes (Dutrillaux and Couturier 1983; Nash et al. 1998; Nash and O'Brien 1987; Wurster‐Hill and Centerwall 1982).

Perissodactyla, consisting of odd‐toed ungulates like horses and rhinos, exhibit distinct patterns of chromosomal evolution. The range of chromosome numbers in this group varies considerably, with values ranging from 2*n* = 32 in the mountain zebra ( *Equus zebra* ; Heinichen 1969) to 2*n* = 84 in the black rhinoceros ( *Diceros bicornis* ; Hungerford et al. 1967). The perissodactyl PAK is thought to have contained 36–38 pairs of autosomes, with a chromosome number of 2*n* = 74–78. CCP has revealed numerous homologies and rearrangements that have shaped the karyotypes of modern perissodactyls (Trifonov et al. 2008) and significantly differing rates of karyotype evolution between equids (horses, donkeys and zebras) and ceratomorphs (tapirs and rhinos).

The domestic horse ( *E. caballus* , 2*n* = 64) and donkey ( *E. asinus* , 2*n* = 62) provide the classic vertebrate example of hybridization between species with different chromosome numbers. Their hybrid offspring, mules and hinnies, inherit an odd chromosome number (2*n* = 63) and are essentially sterile because the unpaired chromosome cannot segregate correctly at meiosis—a strong postzygotic barrier arising directly from chromosome‐number mismatch rather than genic incompatibility. More broadly, the wide range of diploid chromosome numbers among *Equus* species is used routinely as a cytotaxonomic character to distinguish horses, asses and the various zebra species, and is thought to underlie the reduced fertility documented among interspecific equid hybrids in general (Trifonov et al. 2008).

Molecular and morphological studies place Artiodactyla and Cetacea into the order Cetartiodactyla (Price et al. 2005). This group comprises ten extant families including well‐studied Bovidae, Cervidae and Suidae. Chromosome numbers in the order vary from 2*n* = 6 in females and 7 in males ( *M. muntjak* ; Wurster and Benirschke 1970) to 2*n* = 74 in the Camelidae family (O'Brien et al. 2005). Chromosome number 2*n* = 6, 7 represents the lowest recorded among mammals (Wurster and Benirschke 1970), and indeed across all vertebrates (Knytl et al. 2026). Chromosome painting with human and dromedary probes has been used to create genome‐wide comparative maps for species within Cetartiodactyla, including the pilot whale, hippopotamus, okapi and Siberian musk deer (Balmus et al. 2007; Kulemzina et al. 2009). These maps have helped reconstruct the evolutionary pathways and rates of chromosomal rearrangement in Cetartiodactyla.

Cetartiodactyla also provides one of the clearest examples in any vertebrate group of hybridization between species with vastly different chromosome numbers. The Indian muntjac ( *M. muntjak* , 2*n* = 6/7) and the Chinese muntjac ( *M. reevesi* , 2*n* = 46) are morphologically similar and ecologically overlapping, yet differ by several chromosomal fusion events. Despite this extreme karyotypic disparity, the two species can interbreed to produce viable but sterile hybrid offspring (Fontana and Rubini 1990). This postzygotic outcome is more restrictive than the fertile F1 and F2 hybrids documented between the karyotypically divergent crocodilians *C. siamensis* and *C. porosus* (Section 2.1), underscoring that the reproductive consequences of chromosomal divergence are lineage‐specific rather than a fixed function of the size of the chromosome‐number difference itself. The muntjac system nonetheless shows that large‐scale chromosomal repatterning can accumulate between closely related species without immediately abolishing hybrid viability, even as it eventually drives reproductive isolation and provides a good cytotaxonomic marker distinguishing muntjac species from each other.

The cetartiodactyl PAK likely contained 25–26 pairs of autosomes, with a chromosome number of 2*n* = 52–54 (Kulemzina et al. 2009). A cytogenetic signature uniting non‐camelid cetartiodactyls is the association of HSA 8/9. The evolution of cetartiodactyl karyotypes is characterized by alternating periods of low and high rates of chromosomal rearrangements, with the highest rates observed in the Suina lineage and the lowest in cetaceans (Kulemzina et al. 2009).

The rates of chromosomal evolution in Laurasiatheria vary widely among different orders and families. In Perissodactyla, for instance, the rate of chromosomal rearrangements has been relatively low, with notable exceptions in the equid lineage, where rapid karyotypic evolution has been observed. Equids, particularly zebras, exhibit some of the highest rates of chromosomal rearrangement among non‐rodent mammals, driven by factors such as meiotic drive and the rapid evolution of repetitive DNA (Trifonov et al. 2008).

Because rapid rearrangement of this kind is thought to promote reproductive isolation among incipient lineages before genic divergence accumulates, the equid and muntjac examples above illustrate how the tempo of karyotype change can translate into elevated rates of speciation within Laurasiatheria, and how cytogenetics can serve as a practical taxonomic tool wherever morphological differentiation fails. Chromosomal races and hybrid zones are also documented in Laurasiatheria, notably in the common shrew (*Sorex araneus*; Section 6).

### 4.6 Euarchontoglires

Euarchontoglires is the most species‐rich superorder of placental mammals and includes Euarchonta (orders Primates, Dermoptera and Scandentia) and Glires (orders Rodentia and Lagomorpha). The ancestral karyotype of this group is very similar to the ancestral eutherian karyotype (Beklemisheva et al. 2011; Damas et al. 2022), and each order contains extant species with conserved chromosomal structures. The human karyotype is particularly conserved and closely resembles the inferred ancestral eutherian karyotype, making it a valuable reference for many comparative genomic studies. Rodents, which represent the largest mammalian order, show a wide range of karyotypic evolution—from the slowly evolving Sciurimorpha, Castorimorpha and Hystricomorpha (Beklemisheva et al. 2011) to the highly rearranged genomes of muroid rodents (Romanenko et al. 2012).

Diploid chromosome numbers in this group vary from 10 to 102 mostly due to unprecedented diversity of muroid karyotypes, in which the rates of evolution were extremely high (Romanenko et al. 2012). The group is also characterized by the presence of many species with B chromosomes (Vujošević et al. 2018) and by the appearance of groups that overcame ‘the evolutionary trap’ of conserved and divergent sex chromosomes and evolved novel sex determination systems (Saunders and Veyrunes 2021).

Rodents also provide the best‐documented mammalian examples of intraspecific hybridization among chromosomal cytotypes. In the western house mouse ( *Mus musculus domesticus* ), more than 100 geographically distinct chromosomal races distinguished by different combinations of Robertsonian fusions have been described across Europe and numerous narrow hybrid zones exist where adjacent races meet and interbreed (Piálek et al. 2005). Within these zones, structural heterozygotes typically show reduced fertility due to meiotic non‐disjunction in the fused chromosomes. The width and stability of individual zones are used to estimate the strength of selection acting against these hybrids. Consequently, the house mouse serves as a central model for testing chromosomal models of speciation, mirroring the significance of the *S. grammicus* hybrid zone (Section 2.2). Together, the house mouse races and the highly rearranged karyotypes of muroid rodents show that karyotypic differentiation in Euarchontoglires spans a continuum from freely interbreeding chromosomal polymorphisms within a species to cytotaxonomically diagnostic differences between species, consistent with the canalization model of karyotypic adaptation discussed in Section 6.

## 5 Putative Ancestral Amniote Karyotype

The intersection of high‐resolution comparative genomics and molecular cytogenetics has revolutionized our ability to reconstruct the PAK of amniotes. By mapping conserved syntenic blocks and characterizing evolutionary breakpoint regions across diverse lineages, researchers have proposed various architectural models for the ancestral amniote genome, with great variation of diploid chromosome numbers ranging from 48 to 98 (Liu and Zhou 2025; Nakatani et al. 2007; Sacerdot et al. 2018; Uno et al. 2012). The most recent model suggests a highly fragmented ancestral state of 2*n* = 88, characterized by 15 macro‐ and 29 microchromosome pairs (Liu and Zhou 2025). In contrast, the ancestral reptilian (including avian) karyotype is hypothesized to have been more consolidated at 2*n* = 72 (16 macro‐ and 20 microchromosome pairs; Liu and Zhou 2025). Such disparate reconstructions highlight the persistence of significant ‘dark regions’ in our understanding of chromosomal history, where rapid turnover and convergent evolution often mask ancestral states. As we move toward a more comprehensive vertebrate tree of life, it is becoming clear that the ancestral amniote genome was far more complex and fragmented than early models suggested. Reconciling these ~350‐million‐year‐old structural strata remains one of the most elusive puzzles for vertebrate evolutionary cytogenomics.

## 6 The Integrated Tempo of Amniote Chromosomal Evolution

The evolution of amniote genomes is marked by a fascinating dichotomy between extreme chromosomal conservatism and rapid structural reshuffling. While lineages such as birds and many turtles have maintained remarkably stable karyotypes for tens of millions of years, other groups, including many mammals and squamate reptiles, exhibit high rates of chromosomal turnover (Clemente et al. 2021; Mezzasalma et al. 2024; Romanenko et al. 2012; Zhang et al. 2014). Understanding the drivers of this variation requires an integrated look at internal genomic mechanisms and external environmental pressures.

Internal drivers of amniote chromosomal evolution (summarized in Box 1) are often attributed to the landscape of repetitive DNA. In mammals, the expansion of transposable elements, such as Long Interspersed Nuclear Elements (LINEs) and Short Interspersed Nuclear Elements (SINEs), provides substrates for Non‐Allelic Homologous Recombination (NAHR). This process frequently leads to microrearrangements, deletions and duplications. In birds, despite a generally low content of transposable elements, this apparent stability hides substantial lineage‐specific variation: chicken repeat 1 (CR1) retrotransposon activity differs sharply among avian orders, with recent bursts of expansion documented in kiwis, geese and Amazon parrots in contrast to the near‐inactivity in the cassowary and emu (Galbraith et al. 2021). This pattern of lineage‐specific retrotransposon activity crucially contributes to the uneven distribution of karyotypic turnover described through Sections 3.1, 3.3. Meanwhile, the elevated recombination rates of microchromosomes make them hotspots for structural change and their high gene density means such changes are more likely to have evolutionary consequences (Hagen et al. 2020). Hybridization can also destabilize the genome directly. In interspecific marsupial hybrids (e.g., Macropodidae), hybridization triggers retroelement reactivation, genome‐wide undermethylation and de novo chromosome remodelling. This resembles hybrid dysgenesis and offers a plausible mechanistic link between the karyotypically divergent crosses documented in *Petrogale* (Section 4.2) and the genomic instability such crosses can generate (O'Neill et al. 1998). These fragile sites, potentially associated with evolutionary breakpoint regions in the amniote genome, are not randomly distributed (Glugoski et al. 2023; Ruiz‐Herrera and Robinson 2007); they often coincide with regions of high transcriptional activity, where DNA is more accessible and prone to double‐strand breaks, providing the raw material for structural innovation (Deon et al. 2022).

BOX 1 **Mechanisms facilitating chromosomal evolution in amniotes.**

**Table:** 

Mechanism	Definition	Where discussed in this manuscript
Canalization model	Karyotypic change as a primary response to environmental transition, after which molecular/phenotypic innovation occurs	Section 6
Centromere repositioning	A new centromere forms at a different location without a change in gene order	Gavialidae FN differences (2.1); avian Z chromosome (3)
Chromosomal inversion (pericentric/paracentric)	Reversal of a chromosome segment's orientation, with or without the centromere	*Sceloporus grammicus* hybrid zone (2.2); avian Z chromosome (3)
		
Chromosome fusion/fission	Joining of two chromosomes into one (decreases 2*n*) or splitting of one into two (increases 2*n*)	For example, house mouse (4.6); Neoaves (3.3); Chelonians (2.3); crocodilians (2.1)
Genetic hitchhiking	Recombination suppression spreads from a differentiating sex chromosome into adjacent autosomal regions	Section 6 (common shrew)
Hybrid dysgenesis	Genome‐wide destabilization triggered by interspecific hybridization	Section 6; *Petrogale* (4.2)
		
Meiotic drive	Non‐Mendelian transmission bias favouring one chromosome variant, accelerating fixation of new rearrangements	Equids (4.5)
Non‐Allelic Homologous Recombination (NAHR)	Recombination between similar but non‐allelic repeat sequences	Section 6
Ploidy changes	Polyploidization by hybridization (allotriploidy) and ploidy mosaicism (cells of different ploidy within an individual)	Squamates (2.2); *Platemys platycephala* (2.3)
		
Position effect	A rearrangement relocates a gene relative to its regulatory elements, altering expression—a consequence of rearrangement	Section 6
Sex chromosome turnover (incl. sex chromosome–autosome fusion)	Transitions between sex‐determining systems, recruitment of autosomes into sex chromosomes, often coupled with autosomal rearrangements	Section 6; TSD‐GSD transitions (2.2, 2.3); platypus (4.1); swamp wallaby (4.2); avian neo‐sex chromosome (Section 3)
Socially mediated genetic drift	Reduced effective population size increases fixation of rearrangements by drift	Section 6
Chromosome translocation	Transfer of a chromosome segment to a non‐homologous chromosome	Squamates (2.2)
Lienage‐specific transposable element expansion	Episodic proliferation of transposable elements that supplies repeat substrate for NAHR and produces uneven rearrangement rates	Section 6

Sex chromosomes play a disproportionate role in driving the evolution of the entire genome and karyotype (Evans et al. 2024; Kretschmer, Gunski, et al. 2020; Zhou et al. 2021). In amniotes, the transition between different sex‐determining mechanisms, such as the turnover from XY to ZW systems or vice versa or the recruitment of new autosomes into multiple sex chromosome systems (Pšenička et al. 2025; Zhou et al. 2025), can either be triggered by or itself trigger a cascade of autosomal rearrangements. Examples of this sex chromosome‐autosome association can be found in lacertid lizards (squamate reptiles), where a multiple sex chromosome system arises via sex chromosome‐autosome fusion (Mezzasalma et al. 2021). Another compelling example is mammalian *S. araneus* (Eulipotyphla, Laurasiatheria), which shows one of the most remarkable hotspots of intraspecific chromosomal variations in vertebrates, with about 70 described chromosomal races overlapping across numerous hybrid zones in Eurasia (Borodin et al. 2008; White et al. 2010). In *S. araneus* , sex chromosome turnover created an environment favouring autosomal rearrangements and the formation of a multiple sex chromosome (XY_1_Y_2_) system. The suppression of recombination required for sex chromosome differentiation can spread to adjacent autosomal regions via inversions or translocations. This ‘genetic hitchhiking’ of the autosomes can lead to a rapid deviation from the ancestral chromosomal state, particularly in lineages characterized by high rates of speciation (Rice 1987).

While internal molecular mechanisms provide the potential for rearrangement, the fixation of these changes is often driven by ecological factors. In this context, the canalization model (Bickham and Baker 1978) provides an explanation for this trend. The model proposes that karyotypic change is the first response  to environmental transition. Following the establishment of a stable chromosomal set, evolutionary innovation proceeds at the sub‐chromosomal and phenotypic scales (Bickham and Baker 1978). Empirical support for this model, where geographically isolated populations often manifest as distinct karyotypes (karyomorphs), is widespread across the vertebrate tree (Cole 2021; Gvoždik et al. 2024; Lisachov et al. 2018; Orlov et al. 2023; Sember et al. 2015). In amniotes, this is clearly observed in chromosomal races of rodents and lizards, where specific chromosomal configurations correlate with distinct microhabitats or climatic niches. These rearrangements may alter the spatial organization of the genome, bringing distant regulatory elements into proximity with new target genes—a process known as position effect—thereby facilitating rapid phenotypic adaptation to new ecological frontiers (Lewis 1950; Wilson et al. 1990).

Beyond molecular and ecological drivers, social organization, for example kin‐based groups with polygynous harem structures, plays an important role in the fixation of chromosomal rearrangements. By reducing the effective population size and promoting inbreeding, harem systems facilitate genetic drift and the rapid transition of underdominant rearrangements from heterozygosity to homozygosity (Bush et al. 1977). This socially mediated drift explains the high rates of karyotypic turnover observed in diverse amniote lineages, such as equids and rodents (Figure 5; Table S3), providing a behavioural mechanism that supports the canalization model of evolution (sensu Bickham and Baker 1978). Conversely, the relative karyotype stability has been found in harem‐breeding gorillas and specific muroid rodents (Figure 5; Table S3), indicating that social structure is not a universal driver of karyotype diversity. Such discrepancies indicate that the impact of social organization is likely contingent upon effective population size and lineage‐specific context, further modulated by other genomic or life‐history factors (Bush et al. 1977), underscoring the need for further investigation.

Amniote karyotype divergence evidenced in this study is not merely a transient state; it can serve as a primary step toward establishing reproductive isolation and ultimately resulting in allopatric speciation. These structural changes may act as a ‘lock’, preserving the adaptive genetic combinations of a population and canalizing the evolutionary trajectory of a given lineage.

## 7 Conclusions

Here, we present a comprehensive summary of chromosome numbers across amniote vertebrates, including an overview of ancestral karyotypes and sex chromosome systems. The evolution of amniote chromosomes is not a purely stochastic process but a regulated response to both internal genomic mechanisms and external environmental shifts. These same chromosomal dynamics carry direct consequences for lineage diversification, providing both a mechanism of postzygotic reproductive isolation and a practical cytotaxonomic tool across amniote clades. Whether through the slow accumulation of microinversions in birds or the rapid fusions seen in certain mammalian and squamate lineages, chromosomal rearrangements serve as a fundamental engine of amniote biodiversity. By integrating the canalization model and position effect, lineage‐specific transposable element activity and hybridization‐driven genomic instability (Box 1) into our understanding of these dynamics, we can better appreciate the chromosome not just as a static carrier of genetic information, but as a dynamic participant in the adaptation and diversification of life on land.

By bridging the gap between the non‐amniote (Knytl et al. 2026) and amniote (this study) vertebrates, this synthesis provides a broad perspective on the architectural evolution of the vertebrate karyotype. From the early‐diverging lineages of agnathans to the complex genomes of mammals, the two studies examine the tempo and modes of chromosomal change across orders and families, offering an updated understanding of the genomic reshuffling that has driven ~500 My of vertebrate evolutionary history.

## Author Contributions

Data and database source M.K., T.M.D., D.P., V.T., L.V.M. and M.M.; Graphic design M.K., T.M.D. and M.M.; Formal analysis and Software M.K.; Funding acquisition M.K., T.M.D., V.T., F.M.C.S., M.R.V. and M.B.C.; Conceptualization of the study M.K. and M.M.; Visualization M.K.; Data curation and Validation M.K.; Project administration M.K.; Writing – original draft preparation: non‐avian reptiles M.M., birds T.M.D, mammals D.P. and V.T., discussion and final synthesis M.K.; Supervision M.K. and M.M.; All authors reviewed, edited, and approved the final version of the manuscript.

## Funding

This work is supported by the P JAC project CZ.02.01.01/00/22_010/0002902 MSCA Fellowships CZ–UK (M.K.); Conselho Nacional de Desenvolvimento Científico e Tecnológico (CNPq/Brasil): fellowships 150298/2022‐6 (T.M.D.), 313566/2023‐2 (M.R.V), 302928/2021‐9 (M.B.C) and 200247/2025‐5 (F.M.C.S); the Marie Skłodowska‐Curie Actions—COFUND project co‐funded by the European Union, MERIT—Grant Agreement No. 101081195 (V.T.).

## Ethics Statement

No experimental animals were used within the study.

## Conflicts of Interest

The authors declare no conflicts of interest.

## Supporting information


**Table S1:** Chromosome number variation and ancestral karyotype reconstructions in non‐avian Reptilia. Diploid number (2*n*) ranges and putative ancestral chromosome counts are provided for individual families. Chromosome numbers resulting from polyploidy are indicated by the corresponding ploidy level (3×, 4×) in parentheses. Biological (*n*) and evolutionary (*x*) ploidy: the symbol *n* denotes the number of chromosomes in the gametes of an extant species, while *x* represents the number of chromosomes in the gametes of its most recent common ancestor. Families for which no karyotypic data are currently available (Alopoglossidae, Cadeidae, Gerrhopilidae, Anomalepidae, Aniliidae, Anomochilidae, Uropeltidae, Xenophidiidae, Bolyeridae, Pareidae, Homalopsidae, Micrelapidae and Cyclocoridae) have been omitted. Data were compiled from [1–5] and the references cited therein.
**Table S2:** Chromosome evolution in Aves. Diploid chromosome number variation and putative ancestral chromosome numbers are shown for individual orders. No polyploidy was documented across avian taxa. No karyotype data are available for Mesitornithiformes, Phaethontiformes and Leptosomiformes, which have been excluded from the table. Information was taken from [6].
**Table S3:** Chromosomal evolution and ancestral karyotype reconstructions in Mammalia. Karyotypic diversity (2*n*) and putative ancestral chromosome counts are provided for individual mammalian orders. Data were synthesized from references [7–24]. No instances of viable polyploidy were identified within the surveyed taxa.

## Data Availability

All the data supporting the findings of this study are available within the article and its Supporting Information.

## References

[mec70608-bib-0001] Alam, S. M. I. , S. D. Sarre , D. Gleeson , A. Georges , and T. Ezaz . 2018. “Did Lizards Follow Unique Pathways in Sex Chromosome Evolution?” Genes 9, no. 5: 239. 10.3390/genes9050239. PMC5977179 29751579

[mec70608-bib-0002] Altmanová, M. , M. Rovatsos , L. Kratochvíl , and M. Johnson Pokorná . 2016. “Minute Y Chromosomes and Karyotype Evolution in Madagascan Iguanas (Squamata: Iguania: Opluridae).” Biological Journal of the Linnean Society 118, no. 3: 618–633. 10.1111/bij.12751.

[mec70608-bib-0003] Asher, R. J. 2018. “7. Diversity and Relationships Within Crown Mammalia.” Mammalian Evolution, Diversity and Systematics: 301–352. 10.1515/9783110341553-007.

[mec70608-bib-0004] Azevedo, N. F. , M. Svartman , A. Manchester , N. De Moraes‐Barros , R. Stanyon , and A. M. Vianna‐Morgante . 2012. “Chromosome Painting in Three‐Toed Sloths: A Cytogenetic Signature and Ancestral Karyotype for Xenarthra.” BMC Evolutionary Biology 12, no. 1: 36. 10.1186/1471-2148-12-36. PMC3383463 22429690

[mec70608-bib-0005] Balmus, G. , V. A. Trifonov , L. S. Biltueva , et al. 2007. “Cross‐Species Chromosome Painting Among Camel, Cattle, Pig and Human: Further Insights Into the Putative Cetartiodactyla Ancestral Karyotype.” Chromosome Research 15, no. 4: 499–514. 10.1007/s10577-007-1154-x. 17671843

[mec70608-bib-0006] Baron, M. G. , D. B. Norman , and P. M. Barrett . 2017. “A New Hypothesis of Dinosaur Relationships and Early Dinosaur Evolution.” Nature 543, no. 7646: 501–506. 10.1038/nature21700. 28332513

[mec70608-bib-0007] Beklemisheva, V. R. , S. A. Romanenko , L. S. Biltueva , et al. 2011. “Reconstruction of Karyotype Evolution in Core Glires. I. The Genome Homology Revealed by Comparative Chromosome Painting.” Chromosome Research 19, no. 4: 549–565. 10.1007/s10577-011-9210-y. 21559983

[mec70608-bib-0008] Bernardi, G. 1993. “The Vertebrate Genome: Isochores and Evolution.” Molecular Biology and Evolution 10, no. 1: 186–204. 10.1093/oxfordjournals.molbev.a039994. 8450755

[mec70608-bib-0009] Bick, Y. A. E. , and W. D. Jackson . 1967. “A Mammalian X‐O Sex‐Chromosome System in the Monotreme *Tachyglossus aculeatus* Determined From Leucocyte Cultures and Testicular Preparations.” American Naturalist 101, no. 917: 79–86. 10.1086/282472.

[mec70608-bib-0010] Bickham, J. W. , and R. J. Baker . 1978. “Canalization Model of Chromosome Evolution.” American Zoologist 18, no. 3: 600.

[mec70608-bib-0011] Bickham, J. W. , J. J. Bull , and J. M. Legler . 1983. “Karyotypes and Evolutionary Relationships of Trionychoid Turtles.” Cytologia 48, no. 1: 177–183. 10.1508/cytologia.48.177.

[mec70608-bib-0012] Bickham, J. W. , B. G. Hanks , D. W. Hale , and J. E. Martin . 1993. “Ploidy Diversity and the Production of Balanced Gametes in Male Twist‐Necked Turtles ( *Platemys platycephala* ).” Copeia 1993, no. 3: 723. 10.2307/1447233.

[mec70608-bib-0013] Bickham, J. W. , P. K. Tucker , and J. M. Legler . 1985. “Diploid‐Triploid Mosaicism: An Unusual Phenomenon in Side‐Necked Turtles ( *Platemys platycephala* ).” Science 227, no. 4694: 1591–1593. 10.1126/science.227.4694.1591. 17795351

[mec70608-bib-0014] Bininda‐Emonds, O. R. P. , M. Cardillo , K. E. Jones , et al. 2007. “The Delayed Rise of Present‐Day Mammals.” Nature 446, no. 7135: 507–512. 10.1038/nature05634. 17392779

[mec70608-bib-0015] Bista, B. , and N. Valenzuela . 2020. “Turtle Insights Into the Evolution of the Reptilian Karyotype and the Genomic Architecture of Sex Determination.” Genes 11, no. 4: 416. 10.3390/genes11040416. PMC7231036 32290488

[mec70608-bib-0016] Borodin, P. M. , T. V. Karamysheva , N. M. Belonogova , A. A. Torgasheva , N. B. Rubtsov , and J. B. Searle . 2008. “Recombination Map of the Common Shrew, *Sorex araneus* (Eulipotyphla, Mammalia).” Genetics 178, no. 2: 621–632. 10.1534/genetics.107.079665. PMC2248351 18245365

[mec70608-bib-0017] Brochu, C. A. 2003. “Phylogenetic Approaches Toward Crocodylian History.” Annual Review of Earth and Planetary Sciences 31, no. 1: 357–397. 10.1146/annurev.earth.31.100901.141308.

[mec70608-bib-0018] Burgin, C. J. , J. P. Colella , P. L. Kahn , and N. S. Upham . 2018. “How Many Species of Mammals Are There?” Journal of Mammalogy 99, no. 1: 1–14. 10.1093/jmammal/gyx147.

[mec70608-bib-0019] Bush, G. L. , S. M. Case , A. C. Wilson , and J. L. Patton . 1977. “Rapid Speciation and Chromosomal Evolution in Mammals.” Proceedings of the National Academy of Sciences of the United States of America 74, no. 9: 3942–3946. 10.1073/pnas.74.9.3942. PMC431793 269445

[mec70608-bib-0020] Carvalho, C. A. , I. O. Furo , P. C. M. O'Brien , et al. 2021. “Comparative Chromosome Painting in Spizaetus Tyrannus and *Gallus gallus* With the Use of Macro‐ and Microchromosome Probes.” PLoS One 16: e0259905. 10.1371/journal.pone.0259905. PMC8601422 34793511

[mec70608-bib-0021] Casey, A. E. , T. J. Daish , J. L. Barbero , and F. Grützner . 2017. “Differential Cohesin Loading Marks Paired and Unpaired Regions of Platypus Sex Chromosomes at Prophase i.” Scientific Reports 7, no. 1: 4217. 10.1038/s41598-017-04560-5. PMC5484699 28652620

[mec70608-bib-0022] Christidis, L. 1990. “Chordata 3. B. Aves.” In Animal Cytogenetics: 4/3, edited by B. John , 1–116. Gebruder Borntraeger.

[mec70608-bib-0023] Chrostek, G. , A. Domaradzka , A. Yurchenko , L. Kratochvíl , S. Mazzoleni , and M. Rovatsos . 2023. “Cytogenetic Analysis of Seven Species of Gekkonid and Phyllodactylid Geckos.” Genes 14, no. 1: 178. 10.3390/genes14010178. PMC9859368 36672918

[mec70608-bib-0024] Clemente, L. , S. Mazzoleni , E. P. Bellavia , et al. 2020. “Interstitial Telomeric Repeats Are Rare in Turtles.” Genes 11, no. 6: 1–18. 10.3390/genes11060657. PMC7348932 32560114

[mec70608-bib-0025] Clemente, L. , S. Mazzoleni , E. Pensabene , et al. 2021. “Cytogenetic Analysis of the Asian Box Turtles of the Genus Cuora (Testudines, Geoemydidae).” Genes 12, no. 2: 156. 10.3390/genes12020156. PMC7911423 33503936

[mec70608-bib-0026] Cohen, M. M. , and C. Gans . 1970. “The Chromosomes of the Order Crocodilia.” Cytogenetic and Genome Research 9, no. 2: 81–105. 10.1159/000130080. 5461083

[mec70608-bib-0027] Cole, C. J. 2021. “Karyotypes of Coralsnakes (Reptilia: Elapidae) From the Western Hemisphere, With Comments on Intraspecific Variation and Centric Fission of Chromosomes.” American Museum Novitates 2021: 3972. 10.1206/3972.1.

[mec70608-bib-0028] Colston, T. J. , P. Kulkarni , W. Jetz , and R. A. Pyron . 2020. “Phylogenetic and Spatial Distribution of Evolutionary Diversification, Isolation, and Threat in Turtles and Crocodilians (Non‐Avian Archosauromorphs).” BMC Evolutionary Biology 20, no. 1: 81. 10.1186/s12862-020-01642-3. PMC7350713 32650718

[mec70608-bib-0029] Damas, J. , M. Corbo , J. Kim , et al. 2022. “Evolution of the Ancestral Mammalian Karyotype and Syntenic Regions.” Proceedings of the National Academy of Sciences of the United States of America 119, no. 40. 10.1073/pnas.2209139119. PMC9550189 36161960

[mec70608-bib-0030] Damas, J. , J. Kim , M. Farré , D. K. Griffin , and D. M. Larkin . 2018. “Reconstruction of Avian Ancestral Karyotypes Reveals Differences in the Evolutionary History of Macro‐ and Microchromosomes.” Genome Biology 19, no. 1: 1–16. 10.1186/s13059-018-1544-8. PMC6173868 30290830

[mec70608-bib-0031] Damas, J. , R. O'Connor , M. Farré , et al. 2017. “Upgrading Short‐Read Animal Genome Assemblies to Chromosome Level Using Comparative Genomics and a Universal Probe Set.” Genome Research 27, no. 5: 875–884. 10.1101/gr.213660.116. PMC5411781 27903645

[mec70608-bib-0032] de Leo, A. A. , N. Guedelha , R. Toder , et al. 1999. “Comparative Chromosome Painting Between Marsupial Orders: Relationships With a 2*n* = 14 Ancestral Marsupial Karyotype.” Chromosome Research 7, no. 7: 509–517. 10.1023/A:1009233327176. 10598566

[mec70608-bib-0033] de Matos, N. B. , M. Ferreira , F. De Jesus Silva , M. T. Rodrigues , E. S. Da Silva , and C. Garcia . 2016. “Taxonomy and Evolution of Tropidurus (Iguania, Tropiduridae) Based on Chromosomal and DNA Barcoding Analysis.” Journal of Herpetology 50, no. 2: 316–326. 10.1670/13-221.

[mec70608-bib-0034] de Oliveira, E. H. C. , F. A. Habermann , O. Lacerda , I. J. Sbalqueiro , J. Wienberg , and S. Müller . 2005. “Chromosome Reshuffling in Birds of Prey: The Karyotype of the World's Largest Eagle (Harpy Eagle, *Harpia harpyja* ) Compared to That of the Chicken ( *Gallus gallus* ).” Chromosoma 114, no. 5: 338–343. 10.1007/s00412-005-0009-5. 16163545

[mec70608-bib-0035] de Oliveira, E. H. C. , M. M. Tagliarini , M. S. dos Santos , P. C. M. O'Brien , and M. A. Ferguson‐Smith . 2013. “Chromosome Painting in Three Species of Buteoninae: A Cytogenetic Signature Reinforces the Monophyly of South American Species.” PLoS One 8, no. 7: e70071. 10.1371/journal.pone.0070071. PMC3724671 23922908

[mec70608-bib-0036] de Oliveira, E. H. C. , M. M. Tagliarini , J. D. Rissino , et al. 2010. “Reciprocal Chromosome Painting Between White Hawk ( *Leucopternis albicollis* ) and Chicken Reveals Extensive Fusions and Fissions During Karyotype Evolution of Accipitridae (Aves, Falconiformes).” Chromosome Research 18, no. 3: 349–355. 10.1007/s10577-010-9117-z. 20198417

[mec70608-bib-0037] de Oliveira Furo, I. , R. Kretschmer , M. S. Dos Santos , et al. 2017. “Chromosomal Mapping of Repetitive DNAs in Myiopsitta Monachus and *Amazona aestiva* (Psittaciformes, Psittacidae) With Emphasis on the Sex Chromosomes.” Cytogenetic and Genome Research 151, no. 3: 151–160. 10.1159/000464458. 28359066

[mec70608-bib-0038] de Souza, M. S. , S. A. Barcellos , M. d. S. dos Santos , et al. 2022. “Microchromosome BAC‐FISH Reveals Different Patterns of Genome Organization in Three Charadriiformes Species.” Animals 12, no. 21: 3052. 10.3390/ani12213052. PMC9655014 36359176

[mec70608-bib-0039] Deakin, J. E. 2018. “Chromosome Evolution in Marsupials.” Genes 9, no. 2: 72. 10.3390/genes9020072. PMC5852568 29415454

[mec70608-bib-0040] Deakin, J. E. , M. L. Delbridge , E. Koina , et al. 2013. “Reconstruction of the Ancestral Marsupial Karyotype From Comparative Gene Maps.” BMC Evolutionary Biology 13, no. 1: 258. 10.1186/1471-2148-13-258. PMC4222502 24261750

[mec70608-bib-0041] Deakin, J. E. , M. J. Edwards , H. Patel , et al. 2016. “Anchoring Genome Sequence to Chromosomes of the Central Bearded Dragon ( *Pogona vitticeps* ) Enables Reconstruction of Ancestral Squamate Macrochromosomes and Identifies Sequence Content of the Z Chromosome.” BMC Genomics 17, no. 1: 447. 10.1186/s12864-016-2774-3. PMC4902969 27286959

[mec70608-bib-0042] Deakin, J. E. , and T. Ezaz . 2014. “Tracing the Evolution of Amniote Chromosomes.” Chromosoma 123, no. 3: 201–216. 10.1007/s00412-014-0456-y. PMC4031395 24664317

[mec70608-bib-0043] Deakin, J. E. , and T. Ezaz . 2019. “Understanding the Evolution of Reptile Chromosomes Through Applications of Combined Cytogenetics and Genomics Approaches.” Cytogenetic and Genome Research 157, no. 1–2: 7–20. 10.1159/000495974. 30645998

[mec70608-bib-0044] Degrandi, T. M. , S. A. Barcellos , A. L. Costa , A. D. V. Garnero , I. Hass , and R. J. Gunski . 2020. “Introducing the Bird Chromosome Database: An Overview of Cytogenetic Studies in Birds.” Cytogenetic and Genome Research 160, no. 4: 199–205. 10.1159/000507768. 32369809

[mec70608-bib-0045] Degrandi, T. M. , J. C. P. de Oliveira , A. d. A. Soares , et al. 2018. “Karyotype Description and Comparative Analysis in Ringed Kingfisher and Green Kingfisher (Coraciiformes, Alcedinidae).” Comparative Cytogenetics 12, no. 2: 163–170. 10.3897/CompCytogen.v12i2.23883. PMC5958172 29780444

[mec70608-bib-0046] Degrandi, T. M. , A. Del Valle Garnero , P. C. M. O'Brien , et al. 2017. “Chromosome Painting in Trogon s. Surrucura (Aves, Trogoniformes) Reveals a Karyotype Derived by Chromosomal Fissions, Fusions, and Inversions.” Cytogenetic and Genome Research 151, no. 4: 208–215. 10.1159/000471782. 28501862

[mec70608-bib-0047] Deon, G. A. , L. Glugoski , T. Hatanaka , et al. 2022. “Evolutionary Breakpoint Regions and Chromosomal Remodeling in Harttia (Siluriformes: Loricariidae) Species Diversification.” Genetics and Molecular Biology 45, no. 2: 1–12. 10.1590/1678-4685-GMB-2021-0170. PMC9126045 35604463

[mec70608-bib-0048] Dobigny, G. , F. Yang , P. C. M. O'Brien , et al. 2005. “Low Rate of Genomic Repatterning in Xenarthra Inferred From Chromosome Painting Data.” Chromosome Research 13, no. 7: 651–663. 10.1007/s10577-005-1002-9. 16235115

[mec70608-bib-0049] dos Santos, M. d. S. , R. Kretschmer , F. A. O. Silva , et al. 2015. “Intrachromosomal Rearrangements in Two Representatives of the Genus Saltator (Thraupidae, Passeriformes) and the Occurrence of Heteromorphic Z Chromosomes.” Genetica 143, no. 5: 535–543. 10.1007/s10709-015-9851-4. 26092368

[mec70608-bib-0050] dos Santos, M. S. , I. O. Furo , M. M. Tagliarini , et al. 2019. “The Karyotype of the Hoatzin ( *Opisthocomus hoazin* )—A Phylogenetic Enigma of the Neornithes.” Cytogenetic and Genome Research 156, no. 3: 158–164. 10.1159/000494707. 30472715

[mec70608-bib-0051] Dutrillaux, B. , and J. Couturier . 1983. “The Ancestral Karyotype of Carnivora: Comparison With That of Platyrrhine Monkeys.” Cytogenetics and Cell Genetics 35, no. 3: 200–208. 10.1159/000131867. 6861525

[mec70608-bib-0052] Eldridge, M. D. B. , and R. L. Close . 1993. “Radiation of Chromosome Shuffles.” Current Opinion in Genetics & Development 3, no. 6: 915–922. 10.1016/0959-437X(93)90014-G. 8118218

[mec70608-bib-0053] Ellegren, H. 2010. “Evolutionary Stasis: The Stable Chromosomes of Birds.” Trends in Ecology & Evolution 25, no. 5: 283–291. 10.1016/j.tree.2009.12.004. 20363047

[mec70608-bib-0054] Ellegren, H. , L. Smeds , R. Burri , et al. 2012. “The Genomic Landscape of Species Divergence in Ficedula Flycatchers.” Nature 491, no. 7426: 756–760. 10.1038/nature11584. 23103876

[mec70608-bib-0055] Enguito, M. R. C. , A. D. V. Garnero , R. J. Gunski , et al. 2026. “Comparative Chromosomal Analysis of the Z Chromosome in South American Bird Species Shows a High Rate of Intrachromosomal Rearrangements.” Genes 17, no. 1: 112. 10.3390/genes17010112. PMC12841125 41595531

[mec70608-bib-0056] Evans, B. J. , V. Gvoždík , M. Knytl , et al. 2024. “Rapid Sex Chromosome Turnover in African Clawed Frogs (Xenopus) and the Origins of New Sex Chromosomes.” Molecular Biology and Evolution 41, no. 12: 1–20. 10.1093/molbev/msae234. PMC11635168 39665151

[mec70608-bib-0057] Evans, B. J. , N. S. Upham , G. B. Golding , R. A. Ojeda , and A. A. Ojeda . 2017. “Evolution of the Largest Mammalian Genome.” Genome Biology and Evolution 9, no. 6: 1711–1724. 10.1093/gbe/evx113. PMC5569995 28854639

[mec70608-bib-0058] Farré, M. , J. Kim , A. A. Proskuryakova , et al. 2019. “Evolution of Gene Regulation in Ruminants Differs Between Evolutionary Breakpoint Regions and Homologous Synteny Blocks.” Genome Research 29, no. 4: 576–589. 10.1101/gr.239863.118. PMC6442394 30760546

[mec70608-bib-0059] Ferguson‐Smith, M. A. , and V. Trifonov . 2007. “Mammalian Karyotype Evolution.” Nature Reviews Genetics 8, no. 12: 950–962. 10.1038/nrg2199. 18007651

[mec70608-bib-0060] Fontana, F. , and M. Rubini . 1990. “Chromosomal Evolution in Cervidae.” Biosystems 24, no. 2: 157–174. 10.1016/0303-2647(90)90008-O. 2249009

[mec70608-bib-0061] Fornaini, N. R. , H. Černohorská , L. do Vale Martins , and M. Knytl . 2024. “Cytogenetic Analysis of the Fish Genus Carassius Indicates Divergence, Fission, and Segmental Duplication as Drivers of Tandem Repeat and Microchromosome Evolution.” Genome Biology and Evolution 16, no. 3: 1–13. 10.1093/gbe/evae028. PMC11079324 38340334

[mec70608-bib-0062] Fujita, M. K. , S. Singhal , T. O. Brunes , and J. A. Maldonado . 2020. “Evolutionary Dynamics and Consequences of Parthenogenesis in Vertebrates.” Annual Review of Ecology, Evolution, and Systematics 51, no. 1: 191–214. 10.1146/annurev-ecolsys-011720-114900.

[mec70608-bib-0063] Furo, I. d. O. , R. Kretschmer , P. C. O'Brien , et al. 2020. “Chromosomal Evolution in the Phylogenetic Context: A Remarkable Karyotype Reorganization in Neotropical Parrot *Myiopsitta monachus* (Psittacidae).” Frontiers in Genetics 11. 10.3389/fgene.2020.00721. PMC7366516 32754200

[mec70608-bib-0064] Galbraith, J. D. , R. D. Kortschak , A. Suh , and D. L. Adelson . 2021. “Genome Stability Is in the Eye of the Beholder: CR1 Retrotransposon Activity Varies Significantly Across Avian Diversity.” Genome Biology and Evolution 13, no. 12. 10.1093/gbe/evab259. PMC8665684 34894225

[mec70608-bib-0065] Gallardo, M. H. , J. W. Bickham , R. L. Honeycutt , R. A. Ojeda , and N. Köhler . 1999. “Discovery of Tetraploidy in a Mammal.” Nature 401, no. 6751: 341. 10.1038/43815. 10517628

[mec70608-bib-0066] Gamble, T. , T. A. Castoe , S. V. Nielsen , et al. 2017. “The Discovery of XY Sex Chromosomes in a Boa and Python.” Current Biology 27, no. 14: 2148–2153.e4. 10.1016/j.cub.2017.06.010. 28690112

[mec70608-bib-0217] Garnero, A. D. V. , M. A. Ledesma , and R. J. Gunski . 2006. “Alta Homeologia Cariotípica na Família Tinamidae (Aves: Tinamiformes).” Revista Brasileira de Ornitologia 14, no. 24: 53–58.

[mec70608-bib-0067] Gill, F. , D. Donsker , and P. Rasmussen . 2023. “IOC World Bird List: Hummingbirds.” In IOC World Bird List Datasets 13, no. 1. 10.14344/IOC.ML.13.1.

[mec70608-bib-0068] Glas, R. , A. A. De Leo , M. L. Delbridge , et al. 1999. “Chromosome Painting in Marsupials: Genome Conservation in the Kangaroo Family.” Chromosome Research 7, no. 3: 167–176. 10.1023/A:1009291030968. 10421376

[mec70608-bib-0069] Glugoski, L. , G. A. Deon , V. Nogaroto , O. Moreira‐Filho , and M. R. Vicari . 2023. “Robertsonian Fusion Site in *Rineloricaria pentamaculata* (Siluriformes: Loricariidae): Involvement of 5S Ribosomal DNA and Satellite Sequences.” Cytogenetic and Genome Research 162, no. 11–12: 657–663. 10.1159/000530636. 37054691

[mec70608-bib-0070] González, E. J. , M. Martínez‐López , M. A. Morales‐Garduza , R. García‐Morales , P. Charruau , and J. A. Gallardo‐Cruz . 2019. “The Sex‐Determination Pattern in Crocodilians: A Systematic Review of Three Decades of Research.” Journal of Animal Ecology 88, no. 9: 1417–1427. 10.1111/1365-2656.13037. 31286510

[mec70608-bib-0071] Graphodatsky, A. S. , V. A. Trifonov , and R. Stanyon . 2011. “The Genome Diversity and Karyotype Evolution of Mammals.” Molecular Cytogenetics 4, no. 1: 22. 10.1186/1755-8166-4-22. PMC3204295 21992653

[mec70608-bib-0072] Gregory, T. R. 2005. Animal Genome Size Database. http://www.genomesize.com.

[mec70608-bib-0073] Griffin, D. K. , F. Haberman , J. Masabanda , et al. 1999. “Micro‐ and Macrochromosome Paints Generated by Flow Cytometry and Microdissection: Tools for Mapping the Chicken Genome.” Cytogenetics and Cell Genetics 87, no. 3–4: 278–281. 10.1159/000015449. 10702695

[mec70608-bib-0074] Griffin, D. K. , L. B. W. Robertson , H. G. Tempest , and B. M. Skinner . 2007. “The Evolution of the Avian Genome as Revealed by Comparative Molecular Cytogenetics.” Cytogenetic and Genome Research 117, no. 1–4: 64–77. 10.1159/000103166. 17675846

[mec70608-bib-0075] Gunski, R. J. , A. D. Cañedo , A. D. V. Garnero , et al. 2017. “Multiple Sex Chromosome System in Penguins (Pygoscelis, Spheniscidae).” Comparative Cytogenetics 11, no. 3: 541–552. 10.3897/CompCytogen.v11i3.13795. PMC5646662 29093802

[mec70608-bib-0076] Gunski, R. J. , R. Kretschmer , M. S. De Souza , et al. 2019. “Evolution of Bird Sex Chromosomes Narrated by Repetitive Sequences: Unusual W Chromosome Enlargement in *Gallinula melanops* (Aves: Gruiformes: Rallidae).” Cytogenetic and Genome Research 158, no. 3: 152–159. 10.1159/000501381. 31272100

[mec70608-bib-0077] Guttenbach, M. , I. Nanda , W. Feichtinger , J. S. Masabanda , D. K. Griffin , and M. Schmid . 2003. “Comparative Chromosome Painting of Chicken Autosomal Paints 1–9 in Nine Different Bird Species.” Cytogenetic and Genome Research 103, no. 1–2: 173–184. 10.1159/000076309. 15004483

[mec70608-bib-0078] Guyer, M. F. 1990. “Spermatogenesis of Normal and of Hybrid Pigeons [Dissertation, Faculties of the Graduate School of Arts, Literature, and Science].” In Spermatogenesis of Normal and of Hybrid Pigeons: A Dissertation, Submitted to the Faculty of the Graduate School of Arts, Literature, and Science, in Candidacy for the Degree of Doctor of Philosophy. Department of Zoology. 10.5962/bhl.title.155268.

[mec70608-bib-0079] Gvoždik, V. , M. Knytl , A. G. Zassi‐Boulou , N. R. Fornaini , and B. Bergelova . 2024. “Tetraploidy in the Boettger's Dwarf Clawed Frog (Pipidae: *Hymenochirus boettgeri* ) From The Congo Indicates Non‐Conspecificity With the Captive Population.” Zoological Journal of the Linnean Society 200, no. 4: 1034–1047. 10.1093/zoolinnean/zlad119.

[mec70608-bib-0080] Hagen, I. J. , S. Lien , A. M. Billing , et al. 2020. “A Genome‐Wide Linkage Map for the House Sparrow ( *Passer domesticus* ) Provides Insights Into the Evolutionary History of the Avian Genome.” Molecular Ecology Resources 20, no. 2: 544–559. 10.1111/1755-0998.13134. 31912659

[mec70608-bib-0081] Hay, J. M. , S. D. Sarre , D. M. Lambert , F. W. Allendorf , and C. H. Daugherty . 2010. “Genetic Diversity and Taxonomy: A Reassessment of Species Designation in Tuatara (Sphenodon: Reptilia).” Conservation Genetics 11, no. 3: 1063–1081. 10.1007/s10592-009-9952-7.

[mec70608-bib-0082] Hedges, S. B. 2001. “Afrotheria: Plate Tectonics Meets Genomics.” Proceedings of the National Academy of Sciences of the United States of America 98, no. 1: 1–2. 10.1073/pnas.98.1.1. PMC33345 11136239

[mec70608-bib-0083] Heinichen, G. I. 1969. “Karyotype of the South West African Plains Zebra ( *Equus burchelli* Gray, 1824 Subsp.?) and Mountain Zebra ( *Equus zebra hartmannae* Matschie, 1898): Their Cytogenetic Relationship to Chapman's Zebra (Equus Burchelli Antiquorum H. Smith, 1841) and the Cradock.” Madoqua 1: 47–52.

[mec70608-bib-0084] Hill, P. L. , C. P. Burridge , T. Ezaz , and E. Wapstra . 2018. “Conservation of Sex‐Linked Markers Among Conspecific Populations of a Viviparous Skink, Niveoscincus Ocellatus, Exhibiting Genetic and Temperature‐Dependent Sex Determination.” Genome Biology and Evolution 10, no. 4: 1079–1087. 10.1093/gbe/evy042. PMC5905450 29659810

[mec70608-bib-0085] Houck, M. L. , K.‐P. Koepfli , T. Hains , et al. 2023. “Chromosome‐Length Genome Assemblies and Cytogenomic Analyses of Pangolins Reveal Remarkable Chromosome Counts and Plasticity.” Chromosome Research 31, no. 2: 13. 10.1007/s10577-023-09722-y. 37043058

[mec70608-bib-0086] Huang, Z. , I. De O. Furo , J. Liu , et al. 2022. “Recurrent Chromosome Reshuffling and the Evolution of Neo‐Sex Chromosomes in Parrots.” Nature Communications 13, no. 1: 944. 10.1038/s41467-022-28585-1. PMC8854603 35177601

[mec70608-bib-0087] Hungerford, D. A. , H. S. Chandra , and R. L. Snyder . 1967. “Somatic Chromosomes of a Black Rhinoceros ( *Diceros bicornis* Gray 1821).” American Naturalist 101, no. 920: 357–358. 10.1086/282502.

[mec70608-bib-0088] Janes, D. E. , C. L. Organ , M. K. Fujita , A. M. Shedlock , and S. V. Edwards . 2010. “Genome Evolution in Reptilia, the Sister Group of Mammals.” Annual Review of Genomics and Human Genetics 11, no. 1: 239–264. 10.1146/annurev-genom-082509-141646. 20590429

[mec70608-bib-0089] Jarvis, E. D. , S. Mirarab , A. J. Aberer , et al. 2014. “Whole‐Genome Analyses Resolve Early Branches in the Tree of Life of Modern Birds.” Science 346, no. 6215: 1320–1331. 10.1126/science.1253451. PMC4405904 25504713

[mec70608-bib-0090] Jones, M. E. H. 2008. “Skull Shape and Feeding Strategy in Sphenodon and Other Rhynchocephalia (Diapsida: Lepidosauria).” Journal of Morphology 269, no. 8: 945–966. 10.1002/jmor.10634. 18512698

[mec70608-bib-0091] Jorge, W. , D. A. Meritt , and K. Benirschke . 1977. “Chromosome Studies in Edentata.” Cytobios 18, no. 71–72: 157–172. 679723

[mec70608-bib-0092] Joseph, S. , R. E. O'Connor , A. F. Al Mutery , M. Watson , D. M. Larkin , and D. K. Griffin . 2018. “Chromosome Level Genome Assembly and Comparative Genomics Between Three Falcon Species Reveals an Unusual Pattern of Genome Organisation.” Diversity 10, no. 4: 113. 10.3390/d10040113.

[mec70608-bib-0093] Kasai, F. , C. Garcia , M. V. Arruga , and M. A. Ferguson‐Smith . 2003. “Chromosome Homology Between Chicken ( *Gallus gallus* Domesticus) and the Red‐Legged Partridge ( *Alectoris rufa* ); Evidence of the Occurrence of a Neocentromere During Evolution.” Cytogenetic and Genome Research 102, no. 1–4: 326–330. 10.1159/000075770. 14970724

[mec70608-bib-0094] Kasai, F. , P. C. M. O'Brien , and M. A. Ferguson‐Smith . 2013. “Afrotheria Genome; Overestimation of Genome Size and Distinct Chromosome GC Content Revealed by Flow Karyotyping.” Genomics 102, no. 5–6: 468–471. 10.1016/j.ygeno.2013.09.002. 24055950

[mec70608-bib-0218] Knytl, M. , F. M. C. Sassi , M. R. Vicari , et al. 2026. “Chromosome Evolution in Non‐amniote Vertebrates: A Comprehensive Atlas of Karyotype Diversity, Ploidy, and Ancestral States.” Chromosome Research (under review). 10.1111/mec.70608 PMC13743978 42995665

[mec70608-bib-0095] Kretschmer, R. , I. De Oliveira Furo , M. De Bello Cioffi , et al. 2020. “Extensive Chromosomal Fissions and Repetitive DNA Accumulation Shaped the Atypical Karyotypes of Two Ramphastidae (Aves: Piciformes) Species.” Biological Journal of the Linnean Society 130, no. 4: 839–849. 10.1093/BIOLINNEAN/BLAA086.

[mec70608-bib-0096] Kretschmer, R. , M. S. de Souza , I. d. O. Furo , et al. 2021. “Interspecies Chromosome Mapping in Caprimulgiformes, Piciformes, Suliformes, and Trogoniformes (Aves): Cytogenomic Insight Into Microchromosome Organization and Karyotype Evolution in Birds.” Cells 10, no. 4: 826. 10.3390/cells10040826. PMC8067558 33916942

[mec70608-bib-0097] Kretschmer, R. , M. A. Ferguson‐Smith , and E. H. C. de Oliveira . 2018. “Karyotype Evolution in Birds: From Conventional Staining to Chromosome Painting.” Genes 9, no. 4: 181. 10.3390/genes9040181. PMC5924523 29584697

[mec70608-bib-0098] Kretschmer, R. , I. Franz , M. S. de Souza , et al. 2021. “Cytogenetic Evidence Clarifies the Phylogeny of the Family Rhynchocyclidae (Aves: Passeriformes).” Cells 10, no. 10: 2650. 10.3390/cells10102650. PMC8534115 34685630

[mec70608-bib-0099] Kretschmer, R. , R. J. Gunski , A. Del Valle Garnero , et al. 2014. “Molecular Cytogenetic Characterization of Multiple Intrachromosomal Rearrangements in Two Representatives of the Genus Turdus (Turdidae, Passeriformes).” PLoS One 9, no. 7: e103338. 10.1371/journal.pone.0103338. PMC4110018 25058578

[mec70608-bib-0100] Kretschmer, R. , R. J. Gunski , A. d. V. Garnero , et al. 2020. “Chromosomal Analysis in *Crotophaga ani* (Aves, Cuculiformes) Reveals Extensive Genomic Reorganization and an Unusual Z‐Autosome Robertsonian Translocation.” Cells 10, no. 1: 4. 10.3390/cells10010004. PMC7822047 33375072

[mec70608-bib-0101] Kulemzina, A. I. , V. A. Trifonov , P. L. Perelman , et al. 2009. “Cross‐Species Chromosome Painting in Cetartiodactyla: Reconstructing the Karyotype Evolution in Key Phylogenetic Lineages.” Chromosome Research 17, no. 3: 419–436. 10.1007/s10577-009-9032-3. 19350402

[mec70608-bib-0102] Kumar, S. , M. Suleski , J. M. Craig , et al. 2022. “TimeTree 5: An Expanded Resource for Species Divergence Times.” Molecular Biology and Evolution 39, no. 8. 10.1093/molbev/msac174. PMC9400175 35932227

[mec70608-bib-0103] Lahn, B. T. , and D. C. Page . 1999. “Four Evolutionary Strata on the Human X Chromosome.” Science 286, no. 5441: 964–967. 10.1126/science.286.5441.964. 10542153

[mec70608-bib-0104] Lee, L. , E. E. Montiel , B. M. Navarro‐Domínguez , and N. Valenzuela . 2019. “Chromosomal Rearrangements During Turtle Evolution Altered the Synteny of Genes Involved in Vertebrate Sex Determination.” Cytogenetic and Genome Research 157, no. 1–2: 77–88. 10.1159/000497302. 30808820

[mec70608-bib-0105] Lewis, E. B. 1950. “The Phenomenon of Position Effect.” In Advances in Genetics, edited by M. Demerec , 73–115. 10.1016/S0065-2660(08)60083-8. 15425389

[mec70608-bib-0106] Lin, K. W. , and J. Yan . 2008. “Endings in the Middle: Current Knowledge of Interstitial Telomeric Sequences.” Mutation Research‐Reviews in Mutation Research 658, no. 1–2: 95–110. 10.1016/j.mrrev.2007.08.006. 17921045

[mec70608-bib-0107] Lisachov, A. , N. Poyarkov , P. Pawangkhanant , P. Borodin , and K. Srikulnath . 2018. “New Karyotype of Lygosoma Bowringii Suggests Cryptic Diversity.” Herpetology Notes 11: 1083–1088.

[mec70608-bib-0108] Liu, J. , and Q. Zhou . 2025. “Evolution of Chromosomes and Nuclear Architectures of Amniotes.” Nature Communications 17, no. 1: 737. 10.1038/s41467-025-67427-8. PMC12820338 41365915

[mec70608-bib-0109] Machado, C. R. D. , C. Domit , M. B. Pucci , et al. 2020. “Heterochromatin and Microsatellites Detection in Karyotypes of Four Sea Turtle Species: Interspecific Chromosomal Differences.” Genetics and Molecular Biology 43, no. 4: 1–9. 10.1590/1678-4685-GMB-2020-0213. PMC7734918 33270075

[mec70608-bib-0110] Makino, S. 2011. “An Atlas of the Chromosome Numbers in Animals.” In An Atlas of the Chromosome Numbers in Animals. Iowa State College Press. 10.5962/bhl.title.7295.

[mec70608-bib-0111] Mannion, P. D. , A. A. Chiarenza , P. L. Godoy , and Y. N. Cheah . 2019. “Spatiotemporal Sampling Patterns in the 230 Million Year Fossil Record of Terrestrial Crocodylomorphs and Their Impact on Diversity.” Palaeontology 62, no. 4: 615–637. 10.1111/pala.12419.

[mec70608-bib-0112] Marshall Graves, J. A. , and S. Shetty . 2001. “Sex From W to Z: Evolution of Vertebrate Sex Chromosomes and Sex Determining Genes.” Journal of Experimental Zoology 290, no. 5: 449–462. 10.1002/jez.1088. 11555852

[mec70608-bib-0113] Martins, L. F. , E. L. Choueri , A. F. S. Oliveira , et al. 2021. “Whiptail Lizard Lineage Delimitation and Population Expansion as Windows Into the History of Amazonian Open Ecosystems.” Systematics and Biodiversity 19, no. 8: 957–975. 10.1080/14772000.2021.1953185.

[mec70608-bib-0114] Mezzasalma, M. , F. Andreone , W. R. Branch , et al. 2014. “Chromosome Evolution in Pseudoxyrhophiine Snakes From Madagascar: A Wide Range of Karyotypic Variability.” Biological Journal of the Linnean Society 112, no. 3: 450–460. 10.1111/bij.12280.

[mec70608-bib-0115] Mezzasalma, M. , E. Brunelli , G. Odierna , and F. M. Guarino . 2022. “First Insights on the Karyotype Diversification of the Endemic Malagasy Leaf‐Toed Geckos (Squamata: Gekkonidae: Uroplatus).” Animals 12, no. 16: 2054. 10.3390/ani12162054. PMC9404452 36009644

[mec70608-bib-0116] Mezzasalma, M. , E. Brunelli , G. Odierna , and F. M. Guarino . 2023. “Evolutionary and Genomic Diversity of True Polyploidy in Tetrapods.” Animals 13, no. 6: 1033. 10.3390/ani13061033. PMC10044425 36978574

[mec70608-bib-0117] Mezzasalma, M. , F. M. Guarino , and G. Odierna . 2021. “Lizards as Model Organisms of Sex Chromosome Evolution: What We Really Know From a Systematic Distribution of Available Data?” Genes 12, no. 9: 1341. 10.3390/genes12091341. PMC8468487 34573323

[mec70608-bib-0118] Mezzasalma, M. , R. Macirella , G. Odierna , and E. Brunelli . 2024. “Karyotype Diversification and Chromosome Rearrangements in Squamate Reptiles.” Genes 15, no. 3: 371. 10.3390/genes15030371. PMC10970613 38540430

[mec70608-bib-0119] Mezzasalma, M. , J. W. Streicher , F. M. Guarino , et al. 2023. “Microchromosome Fusions Underpin Convergent Evolution of Chameleon Karyotypes.” Evolution 77, no. 9: 1930–1944. 10.1093/evolut/qpad097. 37288542

[mec70608-bib-0120] Montiel, E. E. , D. Badenhorst , L. S. Lee , R. Literman , V. Trifonov , and N. Valenzuela . 2016. “Cytogenetic Insights Into the Evolution of Chromosomes and Sex Determination Reveal Striking Homology of Turtle Sex Chromosomes to Amphibian Autosomes.” Cytogenetic and Genome Research 148, no. 4: 292–304. 10.1159/000447478. 27423490

[mec70608-bib-0121] Moritz, C. 1986. “The Population Biology of Gehyra (Gekkonidae): Chromosome Change and Speciation.” Systematic Biology 35, no. 1: 46–67. 10.1093/sysbio/35.1.46.

[mec70608-bib-0122] Murphy, W. J. , E. Eizirik , S. J. O'Brien , et al. 2001. “Resolution of the Early Placental Mammal Radiation Using Bayesian Phylogenetics.” Science 294, no. 5550: 2348–2351. 10.1126/science.1067179. 11743200

[mec70608-bib-0123] Murphy, W. J. , D. M. Larkin , A. Everts‐Van Der Wind , et al. 2005. “Evolution: Dynamics of Mammalian Chromosome Evolution Inferred From Multispecies Comparative Maps.” Science 309, no. 5734: 613–617. 10.1126/science.1111387. 16040707

[mec70608-bib-0124] Nakatani, Y. , H. Takeda , Y. Kohara , and S. Morishita . 2007. “Reconstruction of the Vertebrate Ancestral Genome Reveals Dynamic Genome Reorganization in Early Vertebrates.” Genome Research 17, no. 9: 1254–1265. 10.1101/gr.6316407. PMC1950894 17652425

[mec70608-bib-0125] Nash, W. G. , J. C. Menninger , H. M. Padilla‐Nash , G. Stone , P. L. Perelman , and S. J. O'Brien . 2008. “The Ancestral Carnivore Karyotype (2*n* = 38) Lives Today in Ringtails.” Journal of Heredity 99, no. 3: 241–253. 10.1093/jhered/esm130. 18339652

[mec70608-bib-0126] Nash, W. G. , and S. J. O'Brien . 1987. “A Comparative Chromosome Banding Analysis of the Ursidae and Their Relationship to Other Carnivores.” Cytogenetics and Cell Genetics 45, no. 3–4: 206–212. 10.1159/000132455. 3691188

[mec70608-bib-0127] Nash, W. G. , J. Wienberg , M. A. Ferguson‐Smith , J. C. Menninger , and S. J. O'brien . 1998. “Comparative Genomics: Tracking Chromosome Evolution in the Family Ursidae Using Reciprocal Chromosome Painting.” Cytogenetics and Cell Genetics 83, no. 3–4: 182–192. 10.1159/000015176. 10072575

[mec70608-bib-0128] Nie, W. , P. C. M. O'Brien , B. L. Ng , et al. 2009. “Avian Comparative Genomics: Reciprocal Chromosome Painting Between Domestic Chicken ( *Gallus gallus* ) and the Stone Curlew ( *Burhinus oedicnemus* , Charadriiformes)—An Atypical Species With Low Diploid Number.” Chromosome Research 17, no. 1: 99–113. 10.1007/s10577-009-9021-6. PMC2697597 19172404

[mec70608-bib-0129] Nie, W. , J. Wang , W. Su , et al. 2012. “Chromosomal Rearrangements and Karyotype Evolution in Carnivores Revealed by Chromosome Painting.” Heredity 108, no. 1: 17–27. 10.1038/hdy.2011.107. PMC3238119 22086079

[mec70608-bib-0130] Nishida, C. , J. Ishijima , A. Kosaka , et al. 2008. “Characterization of Chromosome Structures of Falconinae (Falconidae, Falconiformes, Aves) by Chromosome Painting and Delineation of Chromosome Rearrangements During Their Differentiation.” Chromosome Research 16, no. 1: 171–181. 10.1007/s10577-007-1210-6. 18293111

[mec70608-bib-0131] Nishida‐Umehara, C. , Y. Tsuda , J. Ishijima , et al. 2007. “The Molecular Basis of Chromosome Orthologies and Sex Chromosomal Differentiation in Palaeognathous Birds.” Chromosome Research 15, no. 6: 721–734. 10.1007/s10577-007-1157-7. 17605112

[mec70608-bib-0132] Norris, T. B. , G. K. Rickards , and C. H. Daugherty . 2004. “Chromosomes of Tuatara, Sphenodon, a Chromosome Heteromorphism and an Archaic Reptilian Karyotype.” Cytogenetic and Genome Research 105, no. 1: 93–99. 10.1159/000078014. 15218263

[mec70608-bib-0133] Oaks, J. R. 2011. “A Time‐Calibrated Species Tree of Crocodylia Reveals a Recent Radiation of the True Crocodiles.” Evolution 65, no. 11: 3285–3297. 10.1111/j.1558-5646.2011.01373.x. 22023592

[mec70608-bib-0134] O'Brien, S. J. , J. C. Menninger , and W. G. Nash . 2005. “Atlas of Mammalian Chromosomes.” In Atlas of Mammalian Chromosomes. Wiley‐Liss. 10.2460/ajvr.1978.39.11.1870.

[mec70608-bib-0135] O'Connor, R. E. , L. Kiazim , B. Skinner , et al. 2019. “Patterns of Microchromosome Organization Remain Highly Conserved Throughout Avian Evolution.” Chromosoma 128, no. 1: 21–29. 10.1007/s00412-018-0685-6. PMC6394684 30448925

[mec70608-bib-0136] O'Connor, R. E. , M. N. Romanov , L. G. Kiazim , et al. 2018. “Reconstruction of the Diapsid Ancestral Genome Permits Chromosome Evolution Tracing in Avian and Non‐Avian Dinosaurs.” Nature Communications 9, no. 1: 1–9. 10.1038/s41467-018-04267-9. PMC5962605 29784931

[mec70608-bib-0137] Oguiura, N. , H. Ferrarezzi , and R. F. Batistic . 2010. “Cytogenetics and Molecular Data in Snakes: A Phylogenetic Approach.” Cytogenetic and Genome Research 127, no. 2–4: 128–142. 10.1159/000295789. 20215738

[mec70608-bib-0138] Oliveira, V. C. S. , M. Altmanová , P. F. Viana , et al. 2021. “Revisiting the Karyotypes of Alligators and Caimans (Crocodylia, Alligatoridae) After a Half‐Century Delay: Bridging the Gap in the Chromosomal Evolution of Reptiles.” Cells 10, no. 6: 1397. 10.3390/cells10061397. PMC8228166 34198806

[mec70608-bib-0139] Oliver, P. M. , A. M. Prasetya , L. G. Tedeschi , et al. 2020. “Crypsis and Convergence: Integrative Taxonomic Revision of the *Gehyra australis* Group (Squamata: Gekkonidae) From Northern Australia.” PeerJ 8: e7971. 10.7717/peerj.7971. PMC6991128 32025362

[mec70608-bib-0140] Olmo, E. 2008. “Trends in the Evolution of Reptilian Chromosomes.” Integrative and Comparative Biology 48, no. 4: 486–493. 10.1093/icb/icn049. 21669809

[mec70608-bib-0141] O'Meally, D. , H. Miller , H. R. Patel , J. A. Marshall Graves , and T. Ezaz . 2010. “The First Cytogenetic Map of the Tuatara, *Sphenodon punctatus* .” Cytogenetic and Genome Research 127, no. 2–4: 213–223. 10.1159/000300099. 20299781

[mec70608-bib-0142] O'Neill, R. J. W. , M. J. O'Neill , and J. A. M. Graves . 1998. “Undermethylation Associated With Retroelement Activation and Chromosome Remodelling in an Interspecific Mammalian Hybrid.” Nature 393, no. 6680: 68–72. 10.1038/29985. 9590690

[mec70608-bib-0143] Orlov, V. N. , I. A. Kryshchuk , E. V. Cherepanova , and Y. M. Borisov . 2023. “Channelized Karyotype Evolution of the Common Shrew *Sorex araneus* (Mammalia).” Biology Bulletin Reviews 13, no. 2: 124–139. 10.1134/S2079086423020056.

[mec70608-bib-0144] Ottenburghs, J. 2018. “Exploring the Hybrid Speciation Continuum in Birds.” Ecology and Evolution 8, no. 24: 13027–13034. 10.1002/ece3.4558. PMC6308868 30619602

[mec70608-bib-0145] Pellegrino, K. C. M. , M. T. Rodrigues , and Y. Yonenaga‐Yassuda . 1999. “Chromosomal Polymorphisms due to Supernumerary Chromosomes and Pericentric Inversions in the Eyelidless Microteiid Lizard Nothobachia Ablephara (Squamata, Gymnophthalmidae).” Chromosome Research 7, no. 4: 247–254. 10.1023/A:1009218628942. 10461869

[mec70608-bib-0146] Peng, C. , D.‐D. Wu , J.‐L. Ren , et al. 2023. “Large‐Scale Snake Genome Analyses Provide Insights Into Vertebrate Development.” Cell 186, no. 14: 2959–2976.e22. 10.1016/j.cell.2023.05.030. 37339633

[mec70608-bib-0147] Piálek, J. , H. C. Hauffe , and J. B. Searle . 2005. “Chromosomal Variation in the House Mouse.” Biological Journal of the Linnean Society 84, no. 3: 535–563. 10.1111/j.1095-8312.2005.00454.x.

[mec70608-bib-0148] Pinto, B. J. , T. Gamble , C. H. Smith , and M. A. Wilson . 2023. “A Lizard Is Never Late: Squamate Genomics as a Recent Catalyst for Understanding Sex Chromosome and Microchromosome Evolution.” Journal of Heredity 114, no. 5: 445–458. 10.1093/jhered/esad023. PMC10445521 37018459

[mec70608-bib-0149] Potter, S. , J. G. Bragg , R. Turakulov , et al. 2022. “Limited Introgression Between Rock‐Wallabies With Extensive Chromosomal Rearrangements.” Molecular Biology and Evolution 39, no. 1. 10.1093/molbev/msab333. PMC8788226 34865126

[mec70608-bib-0150] Pozzobon, L. C. , G. A. Toma , M. d. B. Cioffi , E. H. C. de Oliveira , R. Kretschmer , and T. R. O. de Freitas . 2025. “Karyotype Evolution of Suliformes and Description of a ♂Z1Z1Z2Z2/♀Z1Z2W Multiple Sex Chromosome System in Boobies (Sula spp.).” Genome 68: 1–11. 10.1139/gen-2024-0165. 39883916

[mec70608-bib-0151] Price, S. A. , O. R. P. Bininda‐Emonds , and J. L. Gittleman . 2005. “A Complete Phylogeny of the Whales, Dolphins and Even‐Toed Hoofed Mammals (Cetartiodactyla).” Biological Reviews of the Cambridge Philosophical Society 80, no. 3: 445–473. 10.1017/S1464793105006743. 16094808

[mec70608-bib-0152] Pšenička, T. , B. Augstenová , D. Frynta , P. Kornilios , L. Kratochvíl , and M. Rovatsos . 2025. “Sex Chromosome Turnovers and Stability in Snakes.” Molecular Biology and Evolution 42, no. 1. 10.1093/molbev/msae255. PMC11721783 39671568

[mec70608-bib-0153] Reed, K. M. , and J. W. Sites . 1995. “Female Fecundity in a Hybrid Zone Between Two Chromosome Races of the *Sceloporus grammicus* Complex (Sauria, Phrynosomatidae).” Evolution 49, no. 1: 61–69. 10.1111/j.1558-5646.1995.tb05958.x. 28593674

[mec70608-bib-0154] Rens, W. , P. C. M. O'Brien , H. Fairclough , L. Harman , J. A. M. Graves , and M. A. Ferguson‐Smith . 2003. “Reversal and Convergence in Marsupial Chromosome Evolution.” Cytogenetic and Genome Research 102, no. 1–4: 282–290. 10.1159/000075764. 14970718

[mec70608-bib-0155] Rens, W. , P. C. M. O'Brien , J. A. M. Graves , and M. A. Ferguson‐Smith . 2003. “Localization of Chromosome Regions in Potoroo Nuclei ( *Potorous tridactylus* Marsupialia: Potoroinae).” Chromosoma 112, no. 2: 66–76. 10.1007/s00412-003-0246-4. 12844220

[mec70608-bib-0156] Rens, W. , P. C. M. O'Brien , F. Grützner , et al. 2007. “The Multiple Sex Chromosomes of Platypus and Echidna Are Not Completely Identical and Several Share Homology With the Avian Z.” Genome Biology 8, no. 11: R243. 10.1186/gb-2007-8-11-r243. PMC2258203 18021405

[mec70608-bib-0157] Rens, W. , P. C. M. O'Brien , F. Yang , et al. 2001. “Karyotype Relationships Between Distantly Related Marsupials From South America and Australia.” Chromosome Research 9, no. 4: 301–308. 10.1023/A:1016646629889. 11419794

[mec70608-bib-0158] Ribas, T. F. A. , J. C. Pieczarka , D. K. Griffin , et al. 2021. “Analysis of Multiple Chromosomal Rearrangements in the Genome of *Willisornis vidua* Using BAC‐FISH and Chromosome Painting on a Supposed Conserved Karyotype.” BMC Ecology and Evolution 21, no. 1: 34. 10.1186/s12862-021-01768-y. PMC7927240 33653261

[mec70608-bib-0159] Rice, W. R. 1987. “Genetic Hitchhiking and the Evolution of Reduced Genetic Activity of the *Y* Sex Chromosome.” Genetics 116, no. 1: 161–167. 10.1093/genetics/116.1.161. PMC1203114 3596229

[mec70608-bib-0160] Rieseberg, L. H. 2001. “Chromosomal Rearrangements and Speciation.” Trends in Ecology & Evolution 16, no. 7: 351–358. 10.1016/S0169-5347(01)02187-5. 11403867

[mec70608-bib-0161] Robinson, T. J. , B. Fu , M. A. Ferguson‐Smith , and F. Yang . 2004. “Cross‐Species Chromosome Painting in the Golden Mole and Elephant‐Shrew: Support for the Mammalian Clades Afrotheria and Afroinsectiphillia but Not Afroinsectivora.” Proceedings of the Royal Society B: Biological Sciences 271, no. 1547: 1477–1484. 10.1098/rspb.2004.2754. PMC1691750 15306319

[mec70608-bib-0162] Rodionov, A. V. 1996. “Micro vs. Macro: A Review of Structure and Functions of Avian Micro‐ and Macrochromosomes.” Genetika 32, no. 5: 597–608. 8755033

[mec70608-bib-0163] Rodrigues, B. S. , M. D. F. L. de Assis , P. C. M. O'Brien , M. A. Ferguson‐Smith , and E. H. C. De Oliveira . 2014. “Chromosomal Studies on *Coscoroba coscoroba* (Aves: Anseriformes) Reinforce the Coscoroba‐Cereopsis Clade.” Biological Journal of the Linnean Society 111, no. 2: 274–279. 10.1111/bij.12202.

[mec70608-bib-0164] Romanenko, S. A. , P. L. Perelman , V. A. Trifonov , and A. S. Graphodatsky . 2012. “Chromosomal Evolution in Rodentia.” Heredity 108, no. 1: 4–16. 10.1038/hdy.2011.110. PMC3238120 22086076

[mec70608-bib-0165] Román‐Palacios, C. , C. A. Medina , S. H. Zhan , and M. S. Barker . 2021. “Animal Chromosome Counts Reveal a Similar Range of Chromosome Numbers but With Less Polyploidy in Animals Compared to Flowering Plants.” Journal of Evolutionary Biology 34, no. 8: 1333–1339. 10.1111/jeb.13884. 34101952

[mec70608-bib-0166] Rovatsos, M. , M. Altmanová , M. J. Pokorná , P. Velenský , A. S. Baca , and L. Kratochvíl . 2017. “Evolution of Karyotypes in Chameleons.” Genes 8, no. 12: 382. 10.3390/genes8120382. PMC5748700 29231849

[mec70608-bib-0167] Ruiz‐Herrera, A. , and T. J. Robinson . 2007. “Chromosomal Instability in Afrotheria: Fragile Sites, Evolutionary Breakpoints and Phylogenetic Inference From Genome Sequence Assemblies.” BMC Evolutionary Biology 7, no. 1: 199. 10.1186/1471-2148-7-199. PMC2211313 17958882

[mec70608-bib-0168] Sacerdot, C. , A. Louis , C. Bon , C. Berthelot , and H. Roest Crollius . 2018. “Chromosome Evolution at the Origin of the Ancestral Vertebrate Genome.” Genome Biology 19, no. 1: 166. 10.1186/s13059-018-1559-1. PMC6193309 30333059

[mec70608-bib-0169] Saunders, P. A. , and F. Veyrunes . 2021. “Unusual Mammalian Sex Determination Systems: A Cabinet of Curiosities.” Genes 12, no. 11: 1770. 10.3390/genes12111770. PMC8617835 34828376

[mec70608-bib-0170] Schmid, M. , W. Feichtinger , I. Nanda , et al. 1994. “An Extraordinarily Low Diploid Chromosome Number in the Reptile *Gonatodes taniae* (Squamata, Gekkonidae).” Journal of Heredity 85, no. 4: 255–260. 10.1093/oxfordjournals.jhered.a111452. 7930497

[mec70608-bib-0171] Sember, A. , J. Bohlen , V. Šlechtová , M. Altmanová , R. Symonová , and P. Ráb . 2015. “Karyotype Differentiation in 19 Species of River Loach Fishes (Nemacheilidae, Teleostei): Extensive Variability Associated With rDNA and Heterochromatin Distribution and Its Phylogenetic and Ecological Interpretation.” BMC Evolutionary Biology 15, no. 1: 1–22. 10.1186/s12862-015-0532-9. PMC4647339 26573692

[mec70608-bib-0172] Shibusawa, M. , M. Nishibori , C. Nishida‐Umehara , et al. 2004. “Karyotypic Evolution in the Galliformes: An Examination of the Process of Karyotypic Evolution by Comparison of the Molecular Cytogenetic Findings With the Molecular Phylogeny.” Cytogenetic and Genome Research 106, no. 1: 111–119. 10.1159/000078570. 15218250

[mec70608-bib-0173] Shibusawa, M. , C. Nishida‐Umehara , M. Tsudzuki , J. Masabanda , D. K. Griffin , and Y. Matsuda . 2004. “A Comparative Karyological Study of the Blue‐Breasted Quail ( *Coturnix chinensis* , Phasianidae) and California Quail ( *Callipepla californica* , Odontophoridae).” Cytogenetic and Genome Research 106, no. 1: 82–90. 10.1159/000078569. 15218246

[mec70608-bib-0174] Sigeman, H. , S. Ponnikas , and B. Hansson . 2020. “Whole‐Genome Analysis Across 10 Songbird Families Within Sylvioidea Reveals a Novel Autosome–Sex Chromosome Fusion.” Biology Letters 16, no. 4: 20200082. 10.1098/rsbl.2020.0082. PMC7211462 32315592

[mec70608-bib-0175] Sigeman, H. , M. Strandh , E. Proux‐Wéra , et al. 2021. “Avian Neo‐Sex Chromosomes Reveal Dynamics of Recombination Suppression and W Degeneration.” Molecular Biology and Evolution 38, no. 12: 5275–5291. 10.1093/molbev/msab277. PMC8662655 34542640

[mec70608-bib-0176] Sigeman, H. , H. Zhang , S. Ali Abed , and B. Hansson . 2022. “A Novel Neo‐Sex Chromosome in *Sylvietta brachyura* (Macrosphenidae) Adds to the Extraordinary Avian Sex Chromosome Diversity Among Sylvioidea Songbirds.” Journal of Evolutionary Biology 35, no. 12: 1797–1805. 10.1111/jeb.14096. PMC10087220 36156325

[mec70608-bib-0177] Simões, T. R. , M. W. Caldwell , and S. E. Pierce . 2020. “Sphenodontian Phylogeny and the Impact of Model Choice in Bayesian Morphological Clock Estimates of Divergence Times and Evolutionary Rates.” BMC Biology 18, no. 1: 191. 10.1186/s12915-020-00901-5. PMC7720557 33287835

[mec70608-bib-0178] Sites, J. W. , N. H. Barton , and K. M. Reed . 1995. “The Genetic Structure of a Hybrid Zone Between Two Chromosome Races of the *Sceloporus grammicus* Complex (Sauria, Phrynosomatidae) in Central Mexico.” Evolution 49, no. 1: 9–36. 10.1111/j.1558-5646.1995.tb05955.x. 28593667

[mec70608-bib-0179] Skinner, B. M. , and D. K. Griffin . 2012. “Intrachromosomal Rearrangements in Avian Genome Evolution: Evidence for Regions Prone to Breakpoints.” Heredity 108, no. 1: 37–41. 10.1038/hdy.2011.99. PMC3238122 22045382

[mec70608-bib-0180] Srikulnath, K. , S. F. Ahmad , W. Singchat , and T. Panthum . 2021. “Why Do Some Vertebrates Have Microchromosomes?” Cells 10, no. 9: 1–33. 10.3390/cells10092182. PMC8466491 34571831

[mec70608-bib-0181] Srikulnath, K. , W. Thapana , and N. Muangmai . 2015. “Role of Chromosome Changes in Crocodylus Evolution and Diversity.” Genomics & Informatics 13, no. 4: 102–111. 10.5808/GI.2015.13.4.102. PMC4742319 26865840

[mec70608-bib-0182] Srikulnath, K. , Y. Uno , C. Nishida , H. Ota , and Y. Matsuda . 2015. “Karyotype Reorganization in the Hokou Gecko ( *Gekko hokouensis* , Gekkonidae): The Process of Microchromosome Disappearance in Gekkota.” PLoS One 10, no. 8: e0134829. 10.1371/journal.pone.0134829. PMC4524605 26241471

[mec70608-bib-0183] Stingo, V. , and L. Rocco . 2001. “Selachian Cytogenetics: A Review.” Genetica 111, no. 1–3: 329–347. 10.1023/A:1013747215866. 11841178

[mec70608-bib-0184] Stockdale, M. T. , and M. J. Benton . 2021. “Environmental Drivers of Body Size Evolution in Crocodile‐Line Archosaurs.” Communications Biology 4, no. 1: 38. 10.1038/s42003-020-01561-5. PMC7790829 33414557

[mec70608-bib-0185] Suh, A. , L. Smeds , and H. Ellegren . 2015. “The Dynamics of Incomplete Lineage Sorting Across the Ancient Adaptive Radiation of Neoavian Birds.” PLoS Biology 13, no. 8: e1002224. 10.1371/journal.pbio.1002224. PMC4540587 26284513

[mec70608-bib-0186] Svartman, M. , G. Stone , and R. Stanyon . 2005. “Molecular Cytogenetics Discards Polyploidy in Mammals.” Genomics 85, no. 4: 425–430. 10.1016/j.ygeno.2004.12.004. 15780745

[mec70608-bib-0187] Svartman, M. , G. Stone , and R. Stanyon . 2006. “The Ancestral Eutherian Karyotype Is Present in Xenarthra.” PLoS Genetics 2, no. 7: 1006–1011. 10.1371/journal.pgen.0020109. PMC1513266 16848642

[mec70608-bib-0188] Toder, R. , R. J. W. O'Neill , J. Wienberg , P. C. M. O'Brien , L. Voullaire , and J. A. Marshall‐Graves . 1997. “Comparative Chromosome Painting Between Two Marsupials: Origins of an XX/XY1Y2 Sex Chromosome System.” Mammalian Genome 8, no. 6: 418–422. 10.1007/s003359900459. 9166586

[mec70608-bib-0189] Trifonov, V. A. , R. Stanyon , A. I. Nesterenko , et al. 2008. “Multidirectional Cross‐Species Painting Illuminates the History of Karyotypic Evolution in Perissodactyla.” Chromosome Research 16, no. 1: 89–107. 10.1007/s10577-007-1201-7. 18293107

[mec70608-bib-0190] Uetz, P. , P. Freed , R. Aguilar , F. Reyes , J. Kudera , and J. Hošek . 2023. The Reptile Database. http://reptile‐database.org/.

[mec70608-bib-0191] Uno, Y. , C. Nishida , H. Tarui , et al. 2012. “Inference of the Protokaryotypes of Amniotes and Tetrapods and the Evolutionary Processes of Microchromosomes From Comparative Gene Mapping.” PLoS One 7, no. 12: 2–13. 10.1371/journal.pone.0053027. PMC3534110 23300852

[mec70608-bib-0192] Valenzuela, N. , and D. C. Adams . 2011. “Chromosome Number and Sex Determination Coevolve in Turtles.” Evolution 65, no. 6: 1808–1813. 10.1111/j.1558-5646.2011.01258.x. 21644965

[mec70608-bib-0193] van Brink, J. M. 1959. “L'expression Morphologique de la Digamétie Chez les Sauropsidés et les Monotrèmes.” Chromosoma 10, no. 1–6: 1–72. 10.1007/BF00396564. 13629774

[mec70608-bib-0194] Veyrunes, F. , P. D. Waters , P. Miethke , et al. 2008. “Bird‐Like Sex Chromosomes of Platypus Imply Recent Origin of Mammal Sex Chromosomes.” Genome Research 18, no. 6: 965–973. 10.1101/gr.7101908. PMC2413164 18463302

[mec70608-bib-0195] Viana, P. F. , T. Ezaz , M. d. B. Cioffi , et al. 2020. “Revisiting the Karyotype Evolution of Neotropical Boid Snakes: A Puzzle Mediated by Chromosomal Fissions.” Cells 9, no. 10: 1–11. 10.3390/cells9102268. PMC7601083 33050432

[mec70608-bib-0196] Viana, P. F. , E. Feldberg , M. B. Cioffi , et al. 2020. “The Amazonian Red Side‐Necked Turtle *Rhinemys rufipes* (Spix, 1824) (Testudines, Chelidae) Has a GSD Sex‐Determining Mechanism With an Ancient XY Sex Microchromosome System.” Cells 9, no. 9: 2088. 10.3390/cells9092088. PMC7563702 32932633

[mec70608-bib-0197] Viana, P. F. , E. Feldberg , F. H. Takagui , S. Menezes , R. C. Vogt , and T. Ezaz . 2022. “Matamatas *Chelus* spp. (Testudines, Chelidae) Have a Remarkable Evolutionary History of Sex Chromosomes With a Long‐Term Stable XY Microchromosome System.” Scientific Reports 12, no. 1: 6676. 10.1038/s41598-022-10782-z. PMC9035145 35461353

[mec70608-bib-0198] Vicari, M. R. , D. P. Bruschi , D. C. Cabral‐De‐mello , and V. Nogaroto . 2022. “Telomere Organization and the Interstitial Telomeric Sites Involvement in Insects and Vertebrates Chromosome Evolution.” Genetics and Molecular Biology 45, no. 3: e20220071. 10.1590/1678-4685-GMB-2022-0071. PMC9693754 36394537

[mec70608-bib-0199] Vilaça, S. T. , F. Maroso , P. Lara , et al. 2023. “Evidence of Backcross Inviability and Mitochondrial DNA Paternal Leakage in Sea Turtle Hybrids.” Molecular Ecology 32, no. 3: 628–643. 10.1111/mec.16773. 36336814

[mec70608-bib-0200] Vujošević, M. , M. Rajičić , and J. Blagojević . 2018. “B Chromosomes in Populations of Mammals Revisited.” Genes 9, no. 10: 487. 10.3390/genes9100487. PMC6210394 30304868

[mec70608-bib-0201] Warren, W. C. , L. D. W. Hillier , J. A. Marshall Graves , et al. 2008. “Genome Analysis of the Platypus Reveals Unique Signatures of Evolution.” Nature 453, no. 7192: 175–183. 10.1038/nature06936. PMC2803040 18464734

[mec70608-bib-0202] Watanabe, A. , A. C. Fabre , R. N. Felice , et al. 2019. “Ecomorphological Diversification in Squamates From Conserved Pattern of Cranial Integration.” Proceedings of the National Academy of Sciences of the United States of America 116, no. 29: 14688–14697. 10.1073/pnas.1820967116. PMC6642379 31262818

[mec70608-bib-0203] Waters, P. D. , H. R. Patel , A. Ruiz‐Herrera , et al. 2021. “Microchromosomes Are Building Blocks of Bird, Reptile, and Mammal Chromosomes.” Proceedings of the National Academy of Sciences of the United States of America 118, no. 45: 1–11. 10.1073/pnas.2112494118. PMC8609325 34725164

[mec70608-bib-0204] Westerman, M. , R. W. Meredith , and M. S. Springer . 2010. “Cytogenetics Meets Phylogenetics: A Review of Karyotype Evolution in Diprotodontian Marsupials.” Journal of Heredity 101, no. 6: 690–702. 10.1093/jhered/esq076. 20581108

[mec70608-bib-0205] White, T. A. , M. Bordewich , and J. B. Searle . 2010. “A Network Approach to Study Karyotypic Evolution: The Chromosomal Races of the Common Shrew ( *Sorex araneus* ) and House Mouse ( *Mus musculus* ) as Model Systems.” Systematic Biology 59, no. 3: 262–276. 10.1093/sysbio/syq004. 20525634

[mec70608-bib-0206] Wilson, C. , H. J. Bellen , and W. J. Gehring . 1990. “Position Effects on Eukaryotic Gene Expression.” Annual Review of Cell Biology 6, no. 1: 679–714. 10.1146/annurev.cb.06.110190.003335. 2275824

[mec70608-bib-0207] Wright, J. W. , and C. H. Lowe . 1968. “Weeds, Polyploids, Parthenogenesis, and the Geographical and Ecological Distribution of All‐Female Species of *Cnemidophorus* .” Copeia 1968, no. 1: 128. 10.2307/1441559.

[mec70608-bib-0208] Wurster, D. H. , and K. Benirschke . 1970. “Indian Muntjac, *Muntiacus muntjak* : A Deer With a Low Diploid Chromosome Number.” Science 168, no. 3937: 1364–1366. 10.1126/science.168.3937.1364. 5444269

[mec70608-bib-0209] Wurster‐Hill, D. H. , and W. R. Centerwall . 1982. “The Interrelationships of Chromosome Banding Patterns in Canids, Mustelids, Hyena, and Felids.” Cytogenetics and Cell Genetics 34, no. 1–2: 178–192. 10.1159/000131806. 7151489

[mec70608-bib-0210] Yang, F. , E. Z. Alkalaeva , P. L. Perelman , et al. 2003. “Reciprocal Chromosome Painting Among Human, Aardvark, and Elephant (Superorder Afrotheria) Reveals the Likely Eutherian Ancestral Karyotype.” Proceedings of the National Academy of Sciences of the United States of America 100, no. 3: 1062–1066. 10.1073/pnas.0335540100. PMC298726 12552116

[mec70608-bib-0211] Yu, H. , Y. Li , W. Han , et al. 2024. “Pan‐Evolutionary and Regulatory Genome Architecture Delineated by an Integrated Macro‐ and Microsynteny Approach.” Nature Protocols 19, no. 6: 1623–1678. 10.1038/s41596-024-00966-4. 38514839

[mec70608-bib-0212] Zhang, G. , C. Li , Q. Li , et al. 2014. “Comparative Genomics Reveals Insights Into Avian Genome Evolution and Adaptation.” Science 346, no. 6215: 1311–1320. 10.1126/science.1251385. PMC4390078 25504712

[mec70608-bib-0213] Zheng, Y. , and J. J. Wiens . 2016. “Combining Phylogenomic and Supermatrix Approaches, and a Time‐Calibrated Phylogeny for Squamate Reptiles (Lizards and Snakes) Based on 52 Genes and 4162 Species.” Molecular Phylogenetics and Evolution 94: 537–547. 10.1016/j.ympev.2015.10.009. 26475614

[mec70608-bib-0214] Zhou, Q. , J. Zhang , D. Bachtrog , et al. 2014. “Complex Evolutionary Trajectories of Sex Chromosomes Across Bird Taxa.” Science 346, no. 6215. 10.1126/science.1246338. PMC6445272 25504727

[mec70608-bib-0215] Zhou, Y. , J. Jin , X. Li , et al. 2025. “Chromosome‐Level Echidna Genome Illuminates Evolution of Multiple Sex Chromosome System in Monotremes.” GigaScience 14. 10.1093/gigascience/giae112. PMC11710854 39778707

[mec70608-bib-0216] Zhou, Y. , L. Shearwin‐Whyatt , J. Li , et al. 2021. “Platypus and Echidna Genomes Reveal Mammalian Biology and Evolution.” Nature 592, no. 7856: 756–762. 10.1038/s41586-020-03039-0. PMC8081666 33408411

