# Supplementary material for: Chromosome Evolution in Amniote Vertebrates: A Comprehensive Atlas of Karyotypic Diversity From Ancestral States to Rapid Turnover
Source: Mol Ecol. 2026 Nov 7;35(21):e70608. doi: 10.1111/mec.70608 (PMC13743978; doi:10.1111/mec.70608)
Supplement: Supplementary file 1 [file MEC-35-e70608-s001.docx]

**Supplementary Materials**

**Chromosome evolution in amniote vertebrates: A comprehensive atlas of karyotypic diversity from ancestral states to rapid turnover**

Martin Knytl^1,2†*^, Tiago Marafiga Degrandi^3^, Dmitry Prokopov^4,5^, Vladimir Trifonov^6^, Lívia do Vale Martins^7,8^, Francisco de Menezes Cavalcante Sassi^9,10^, Marcelo Ricardo Vicari^11^, Marcelo de Bello Cioffi^12^, Marcello Mezzasalma^13†*^

^1^ Department of Cell Biology, Charles University, Viničná 7, Prague, 12843, Czech Republic

^2^ Department of Biology, McMaster University, 1280 Main Street West, Hamilton, L8S4K1, ON, Canada

^3^ Laboratory of Genetics and Evolution, Department of Structural, Molecular Biology and Genetics, State University of Ponta Grossa, Ponta Grossa 84010-330, PR, Brazil

^4^ Centre for Haemato-Oncology, Barts Cancer Institute, Queen Mary University of London, London, UK

^5^ QMUL Centre for Epigenetics, Queen Mary University of London, London, UK

^6^ Institute of Animal Physiology and Genetics CAS, Rumburská 89, 27721 Liběchov, Czech Republic

^7^ Federal University of Piauí, Campus Amilcar Ferreira Sobral, 64808-605, Floriano, Piauí, Brazil

^8^ Post-Graduation Program in Agronomy, Federal University of Piauí, 64049-550, Teresina, Piauí, Brazil

^9^ MOE Key Laboratory of Freshwater Fish Reproduction and Development, School of Life Sciences, Southwest University, Chongqing 400715, China

^10^ Aquaculture Center of UNESP, São Paulo State University, Jaboticabal 14884-900, SP, Brazil

^11^ Department of Structural Biology, Molecular and Genetics, Universidade Estadual de Ponta Grossa, Av. Carlos Cavalcanti, 4748, 84030-900, Ponta Grossa, Paraná, Brazil

^12^ Departmento de Genética e Evolução, Universidade Federal de São Carlos, Rd Washington Luis km 235, 13565-905 São Carlos - SP, Brazil

^13^ Department of Biology, Ecology and Earth Science, University of Calabria, Via P. Bucci 4/B, 87036 Rende, Italy

^†^ Martin Knytl and Marcello Mezzasalma are co-corresponding and co-senior authors

^*^ Correspondence: Martin Knytl [martin.knytl@natur.cuni.cz](mailto:martin.knytl@natur.cuni.cz), Department of Cell Biology, Charles University, Viničná 7, Prague, 12843, Czech Republic; Marcello Mezzasalma [marcello.mezzasalma@unical.it](mailto:marcello.mezzasalma@unical.it), Department of Biology, Ecology and Earth Science, University of Calabria, Via P. Bucci 4/B, 87036 Rende, Italy

| **Clade** | **Family** | **Chromosome number variation (2n)** | **Ancestral chromosome number (2n)** |
| --- | --- | --- | --- |
| Crocodylia | Gavialidae | 32 | 32 |
|  | Alligatoridae | 32, 42 | 32 |
|  | Crocodylidae | 30-34, 38 | 32 |
| Rhynchocephalia | Sphenodontidae | 34 | 34 |
| Squamata | Dibamidae | 36 | 36 |
|  | Carphodactylidae | 38 | 38 |
|  | Pygopodidae | 34-38, 42 | 38 |
|  | Diplodactylidae | 24, 32-38 | 38 |
|  | Eublepharidae | 24, 32, 34, 38 | 38 |
|  | Sphaerodactylidae | 16, 22, 36, 42 |  |
|  | Phyllodactylidae | 32, 34, 38, 40 |  |
|  | Gekkonidae | 28, 32-46, 56, 60, 66 (3x = 56, 60, 66) |  |
|  | Xantusiidae | 24, 32, 36-42 (3x = 42) | 40 |
|  | Gerrhosauridae | 34 | 34 |
|  | Cordylidae | 34, 44, 46 | 34 |
|  | Scincidae | 24-32, 36 |  |
|  | Teiidae | 30, 38, 44-52, 62, 68, 70, 92 (3x = 68, 69, 70, 4x = 92) |  |
|  | Gymnophtalmidae | 32, 40-44, 50, 52, 58, 62-66 (3x = 66) |  |
|  | Rhineuridae | 46 |  |
|  | Bipedidae | 40, 46 |  |
|  | Blanidae | 32 | 32 |
|  | Trogonophiidae | 36 | 36 |
|  | Amphisbaenidae | 30-44 |  |
|  | Lacertidae | 24, 26, 36-42, 76 (3x = 37, 4x = 76) | 40 |
|  | Helodermatidae | 36, 38 | 36 |
|  | Xenosauridae | 36 | 36 |
|  | Diploglossidae | 36 | 36 |
|  | Anniellidae | 20-24, 36 |  |
|  | Anguidae | 30, 36, 44, 48 |  |
|  | Shinisauridae | 32 | 32 |
|  | Lanthanotidae | dubious | dubious |
|  | Varanidae | 40 | 40 |
|  | Chamaeleonidae | 20-38, 64 | 36 |
|  | Agamidae | 28-36, 42-48, 54, 72 (3x ~ 54, 4n ~ 72) | 34-36 |
|  | Leiocephalidae | 34 | 34 |
|  | Iguanidae | 36 | 36 |
|  | Hoplocercidae | 36 | 36 |
|  | Corytophanidae | 36 | 36 |
|  | Crotaphytidae | 36 | 36 |
|  | Tropiduridae | 36 | 36 |
|  | Opluridae | 36 | 36 |
|  | Leiosauridae | 36 | 36 |
|  | Liolaemidae | 30-44, 48 (3x = 48) |  |
|  | Phrynosomatidae | 22-26, 30-36, 40, 46, 47 (3x = 47) |  |
|  | Polychrotidae | 20, 26-30 |  |
|  | Dactyloidae | 24-30, 34, 36, 44, 46 |  |
|  | Leptotyphlopidae | 36 | 36 |
|  | Xenotyphlopidae | 36 | 36 |
|  | Typhlopidae | 30-34, 42 (3x = 42) |  |
|  | Tropidophidae | 34 | 34 |
|  | Cylindrophiidae | 36 | 36 |
|  | Xenopeltidae | 36 | 36 |
|  | Loxocemidae | 36 | 36 |
|  | Pythonidae | 36 | 36 |
|  | Calabaridae | 36 | 36 |
|  | Boidae | 34, 36, 40, 44 | 36 |
|  | Acrochordidae | 36 | 36 |
|  | Xenodermidae | 32 | 32 |
|  | Viperidae | 36, 42 | 36 |
|  | Lamprophiidae | 34-48 |  |
|  | Elapidae | 28-42 |  |
|  | Colubridae | 24, 28-42, 46, 50, 52, 54 (3x = 54) | 36 |
| Pleurodira | Chelidae | 40, 42, 50-60, 64 | 52 |
|  | Podocnemididae | 26, 28 | 32 |
|  | Pelomedusidae | 34, 36 | 36 |
|  | Carettochelyidae | 68 | 68 |
| Cryptodira | Trionychidae | 66 | 66 |
|  | Cheloniidae | 56 | 56 |
|  | Dermochelyidae | 56 | 56 |
|  | Kinosternidae | 54, 56 | 56 |
|  | Dermatemidydae | 56 | 56 |
|  | Chelydridae | 52 | 52 |
|  | Emydidae | 48-52 | 50 |
|  | Platysternidae | 54 | 54 |
|  | Geoemydidae | 50, 52, 56 | 52 |
|  | Testudinidae | 50-54 | 52 |
| **-** | **-** | **-** | **-** |

**Table S1. Chromosome number variation and ancestral karyotype reconstructions in non-avian Reptilia**. Diploid number (2n) ranges and putative ancestral chromosome counts are provided for individual families. Chromosome numbers resulting from polyploidy are indicated by the corresponding ploidy level (3x, 4x) in parentheses. Biological (*n*) and evolutionary (*x*) ploidy refer to the number of chromosome sets present in a nucleus. The symbol *n* denotes the number of chromosomes in the gametes of an extant species, while *x* represents the number of chromosomes in the gametes of its most recent common ancestor. Families for which no karyotypic data are currently available (Alopoglossidae, Cadeidae, Gerrhopilidae, Anomalepidae, Aniliidae, Anomochilidae, Uropeltidae, Xenophidiidae, Bolyeridae, Pareidae, Homalopsidae, Micrelapidae, and Cyclocoridae) have been omitted. Data were compiled from [1–5] and the references cited therein.

| **Clade** | **Order** | **Chromosome number variation (2n)** | **Ancestral chromosome number (2n)** |
| --- | --- | --- | --- |
| Palaeognathae | Struthioniformes (Ostrich) | 80, 92 | 80 |
|  | Tinamiformes (Tinamous) | 78, 80 | 80 |
| Galloanseres | Anseriformes (Waterfowl) | 74, 98 | 80 |
|  | Galliformes (Landfowl) | 50, 66, 70, 74-84, 88-92, 98 | 80 |
| Neoaves | Podicipediformes (Grebes) | 58, 78, 80 | 80 |
|  | Phoenicopteriformes (Flamingos) | 80 | 80 |
|  | Columbiformes (Doves) | 68-80, 86 | 80 |
|  | Pterocliformes (Sandgrouse) | 80 | 80 |
|  | Cuculiformes | 64, 74-80, 90 | 80 |
|  | Musophagiformes | 82, 136-142 | 80 |
|  | Otidiformes (Bustards) | 78, 80 | 80 |
|  | Caprimulgiformes (Nightjars) | 68-78, 82, 86 | 80 |
|  | Caprimulgiformes (Swifts) | 62-66, 70, 78 | 80 |
|  | Caprimulgiformes (Hummingbirds) | 74, 78-82 | 80 |
|  | Opisthocomiformes (Hoatzin) | 80 | 80 |
|  | Gruiformes (Cranes) | 72-82, 92 | 80 |
|  | Charadriiformes (Plovers) | 42, 50, 54, 60, 66-72, 76, 78, 80-94 | 80 |
|  | Eurypygiformes (Sunbittern) | 78, 80 | 80 |
|  | Gaviiformes (Loons) | 88 | 80 |
|  | Sphenisciformes (Penguins) | 68, 72, 78, 92-96 | 80 |
|  | Procellariiformes (Fulmars) | 70, 74, 80 | 80 |
|  | Pelecaniformes (Cormorants) | 62, 68-76, 86 | 80 |
|  | Pelecaniformes (Ibises) | 50, 66-72 | 80 |
|  | Pelecaniformes (Herons) | 60-68, 72, 74 | 80 |
|  | Pelecaniformes (Pelicans) | 66 | 80 |
|  | Accipitriformes (New World Vultures) | 80 | 80 |
|  | Accipitriformes (Eagles) | 54, 58-62, 66, 68, 72, 74, 78-82 | 80 |
|  | Strigiformes | 50, 66, 70, 76, 80-86, 90, 92 | 80 |
|  | Coliiformes | 78 | 80 |
|  | Trogoniformes | 76, 82 | 80 |
|  | Bucerotiformes | 68, 70, 88, 90, 126 | 80 |
|  | Piciformes | 62, 64, 70, 80, 84-98, 102, 106, 108, 112, 114 | 80 |
|  | Coraciiformes | 40, 42, 76, 78, 82, 84, 90, 94, 122, 132 | 80 |
|  | Cariamiformes | 106, 108 | 80 |
|  | Falconiformes | 40, 48, 50, 52, 90, 92 | 80 |
|  | Psittaciformes | 46-50, 54-76, 82, 86, 92 | 80 |
|  | Passeriformes | 56, 60-64, 68-88, 94 | 80 |
| **-** | **-** | **-** | **-** |

**Table S2: Chromosome evolution in Aves**. Diploid chromosome number variation and putative ancestral chromosome numbers are shown for individual orders. No polyploidy was documented across avian taxa. No karyotype data are available for Mesitornithiformes, Phaethontiformes, Leptosomiformes, which have been excluded from the table. Information was taken from [6].

| **Clade** | **Order** | **Chromosome number variation (2n)** | **Ancestral chromosome number (2n)** |
| --- | --- | --- | --- |
| Monotremaformes | Monotremata | 52, 63-64 | 64 |
| Ameridelphia (  Marsupialia) | Didelphimorphia | 14, 18, 22 | 14 |
|  | Paucituberculata | 14 | 14 |
| Australidelphia (  Marsupialia) | Microbiotheria | 13/14 | 14 |
|  | Peramelemorpha | 14, 18/19 | 14 |
|  | Notoryctemorphia | 20 | 20 |
|  | Dasyuromorphia | 14 | 14 |
|  | Diprotodontia | 10-16, 20-24 | 14 |
| Afrotheria | Proboscidea | 56 | 56 |
|  | Sirenia | 48 | 48 |
|  | Hyracoidea | 54 | 54 |
|  | Tubulidentata | 20 | 20 |
|  | Macroscelidea | 26 | 26 |
|  | Afrosoricida | 26, 30-40, 56 | 56 |
| Xenarthra | Cingulata | 38, 50, 58, 60, 64 | 48 |
|  | Pilosa | 50, 53, 54, 60, 66 | 48 |
| Laurasiatheria | Pholidota | 38, 40, 114 | 36-38 |
|  | Carnivora | 30, 35-44, 50-54, 64-66, 72-74, 78 | 38-42 |
|  | Perissodactyla | 32, 44-46, 52-56, 62-66, 80-84 | 74-78 |
|  | Cetartyodactyla | 6-9, 14, 20, 26, 30-62, 66-70, 74 | 50-54 |
|  | Chiroptera | 14-56, 62 | 44 |
|  | Eulipotyphla | 19-37, 40-44, 48-50 | - |
| Euarchontoglires | Primates | 16, 20, 22, 26, 30, 34, 38, 42-54, 58-62, 66, 70-72, 80 | 48-50 |
|  | Dermoptera | 38,56 | 56? |
|  | Scadentia | 62,66 | 62 |
|  | Lagomorpha | 38-44, 48, 54, 68 | 56 |
|  | Rodentia | 10, 15-68, 78-80, 94, 102 | 50 |
| - | - | - | - |

**Table S3: Chromosomal evolution and ancestral karyotype reconstructions in Mammalia**. Karyotypic diversity (2n) and putative ancestral chromosome counts are provided for individual mammalian orders. Data were synthesized from references [7–24]. No instances of viable polyploidy were identified within the surveyed taxa.

**Supplementary references**

1.	Mezzasalma M, Macirella R, Odierna G, Brunelli E. Karyotype Diversification and Chromosome Rearrangements in Squamate Reptiles. Genes (Basel). 2024;15: 371. doi:10.3390/genes15030371

2.	Mezzasalma M, Brunelli E, Odierna G, Guarino FM. Evolutionary and Genomic Diversity of True Polyploidy in Tetrapods. Animals. 2023;13: 1033. doi:10.3390/ani13061033

3.	Mezzasalma M, Guarino FM, Odierna G. Lizards as Model Organisms of Sex Chromosome Evolution: What We Really Know from a Systematic Distribution of Available Data? Genes (Basel). 2021;12: 1341. doi:10.3390/genes12091341

4.	Olmo E, Signorino G. Chromorep: A reptiles chromosomes database. 2005.

5.	Galoyan E, Nazarov R, Altmanová M, Matveevsky S, Kropachev I, Dedukh D, et al. Natural repeated backcrosses lead to triploidy and tetraploidy in parthenogenetic butterfly lizards (Leiolepis: Agamidae). Sci Rep. 2025;15: 3094. doi:10.1038/s41598-024-83300-y

6.	Degrandi TM, Barcellos SA, Costa AL, Garnero ADV, Hass I, Gunski RJ. Introducing the Bird Chromosome Database: An Overview of Cytogenetic Studies in Birds. Cytogenet Genome Res. 2020;160: 199–205. doi:10.1159/000507768

7.	Damas J, Corbo M, Kim J, Turner-Maier J, Farré M, Larkin DM, et al. Evolution of the ancestral mammalian karyotype and syntenic regions. Proceedings of the National Academy of Sciences. 2022;119. doi:10.1073/pnas.2209139119

8.	Beklemisheva VR, Romanenko SA, Biltueva LS, Trifonov VA, Vorobieva N V., Serdukova NA, et al. Reconstruction of karyotype evolution in core Glires. I. The genome homology revealed by comparative chromosome painting. Chromosome Research. 2011;19: 549–565. doi:10.1007/s10577-011-9210-y

9.	Müller S, Stanyon R, O’Brien PCM, Ferguson-Smith MA, Plesker R, Wienberg J. Defining the ancestral karyotype of all primates by multidirectional chromosome painting between tree shrews, lemurs and humans. Chromosoma. 1999;108: 393–400. doi:10.1007/s004120050391

10.	Nie W, Fu B, O’Brien PC, Wang J, Su W, Tanomtong A, et al. Flying lemurs – The “flying tree shrews”? Molecular cytogenetic evidence for a Scandentia-Dermoptera sister clade. BMC Biol. 2008;6: 18. doi:10.1186/1741-7007-6-18

11.	Biltueva L, Vorobieva N. Chromosome Evolution in Eulipotyphla. Cytogenet Genome Res. 2012;137: 154–164. doi:10.1159/000339889

12.	Volleth M, Heller KG. Chromosome number reduction accompanied by extensive heterochromatin addition in the bat &lt;i&gt;Glauconycteris beatrix&lt;/i&gt; (Mammalia; Chiroptera, Vespertilionidae). Cytogenet Genome Res. 2007;119: 245–247. doi:10.1159/000112069

13.	Trifonov VA, Stanyon R, Nesterenko AI, Fu B, Perelman PL, O’Brien PCM, et al. Multidirectional cross-species painting illuminates the history of karyotypic evolution in Perissodactyla. Chromosome Research. 2008;16: 89–107. doi:10.1007/s10577-007-1201-7

14.	Beklemisheva VR, Perelman PL, Lemskaya NA, Kulemzina AI, Proskuryakova AA, Burkanov VN, et al. The Ancestral Carnivore Karyotype As Substantiated by Comparative Chromosome Painting of Three Pinnipeds, the Walrus, the Steller Sea Lion and the Baikal Seal (Pinnipedia, Carnivora). PLoS One. 2016;11: e0147647. doi:10.1371/journal.pone.0147647

15.	Houck ML, Koepfli K-P, Hains T, Khan R, Charter SJ, Fronczek JA, et al. Chromosome-length genome assemblies and cytogenomic analyses of pangolins reveal remarkable chromosome counts and plasticity. Chromosome Research. 2023;31: 13. doi:10.1007/s10577-023-09722-y

16.	Svartman M. Reconstruction of the Ancestral Xenarthran Karyotype. Encyclopedia of Life Sciences. Wiley; 2013. doi:10.1002/9780470015902.a0024972

17.	Deakin JE, Delbridge ML, Koina E, Harley N, Alsop AE, Wang C, et al. Reconstruction of the ancestral marsupial karyotype from comparative gene maps. BMC Evol Biol. 2013;13: 258. doi:10.1186/1471-2148-13-258

18.	Yang F, Alkalaeva EZ, Perelman PL, Pardini AT, Harrison WR, O’Brien PCM, et al. Reciprocal chromosome painting among human, aardvark, and elephant (superorder Afrotheria) reveals the likely eutherian ancestral karyotype. Proceedings of the National Academy of Sciences. 2003;100: 1062–1066. doi:10.1073/pnas.0335540100

19.	Zhou Y, Jin J, Li X, Gedman G, Pelan S, Rhie A, et al. Chromosome-level echidna genome illuminates evolution of multiple sex chromosome system in monotremes. Gigascience. 2025;14. doi:10.1093/gigascience/giae112

20.	Graphodatsky Alexander, Perelman PL., O’Brien SJ. Atlas of mammalian chromosomes. 2nd ed. Wiley Blackwel; 2020. doi:10.1002/9781119418061

21.	Rens W, O’Brien PCM, Fairclough H, Harman L, Graves JAM, Ferguson-Smith MA. Reversal and convergence in marsupial chromosome evolution. Cytogenet Genome Res. 2003;102: 282–290. doi:10.1159/000075764

22.	Makino S. An atlas of the chromosome numbers in animals. Ames: Iowa State College Press; 1951. doi:10.5962/bhl.title.7295

23.	Hösli P, Thürig D. The karyotype of the male African elephant: Loxodonta africana. Mammal Chromo News. 1970;11: 110–112.

24.	Hamada T. Cytogenetic studies on Insectivora, I. Comparison of karyotypes in two species of shrew moles in Japan. La Kromosomo II. 1980;20: 285–290.
